# Trends in Untreated Tuberculosis in Large Municipalities, Brazil, 2008–2017

**DOI:** 10.3201/eid2703.204094

**Published:** 2021-03

**Authors:** Melanie H. Chitwood, Daniele M. Pelissari, Gabriela Drummond Marques da Silva, Patricia Bartholomay, Marli Souza Rocha, Denise Arakaki-Sanchez, Mauro Sanchez, Ted Cohen, Marcia C. Castro, Nicolas A. Menzies

**Affiliations:** Yale School of Public Health, New Haven, Connecticut, USA (M.H. Chitwood, T. Cohen);; Ministry of Health, Brasília, Brazil (D.M. Pelissari, P. Bartholomay, M.S. Rocha, D. Arakaki-Sanchez);; Oswaldo Cruz Foundation, Minas Gerais, Brazil (G.D. Marques da Silva);; University of Brasília, Brasília (M. Sanchez);; Harvard T.H. Chan School of Public Health, Boston, Massachusetts, USA (M.C. Castro, N.A. Menzies)

**Keywords:** tuberculosis and other mycobacteria, Brazil, epidemiology, bacteria

## Abstract

We adapted a mathematical modeling approach to estimate tuberculosis (TB) incidence and fraction treated for 101 municipalities of Brazil during 2008–2017. We found the average TB incidence rate decreased annually (0.95%), and fraction treated increased (0.30%). We estimated that 9% of persons with TB did not receive treatment in 2017.

Many countries that have considerable subnational variation in tuberculosis (TB) burden also have decentralized the management and implementation of control policies. In this context, local estimates of TB burden can convey actionable insights for these TB control decisions. Reported cases are commonly used as a proxy for TB burden; however, reported cases may not reflect the true burden because areas of apparently low burden may instead represent areas of inadequate case detection. Modeling approaches have been proposed to adjust for this bias and enable valid inference of TB incidence, but these approaches typically require primary data collection (*1*,*2*). Alternative methods make use of routinely collected data (*3**–**5*). We applied a recently developed Bayesian method to report unbiased estimates of TB incidence and the completeness of case detection in Brazil’s state capitals and 100 most populous municipalities during 2008–2017 (Appendix). The Office of Human Research Administration at Harvard T.H. Chan School of Public Health reviewed the initial study submission (protocol no. IRB18-0759) and determined that it met the criteria for exemption from ethics board review. 

## The Study

We selected the 100 most populous municipalities in Brazil (on the basis of mean population between 2008–2017) plus Palmas, the 1 state capital that was not among those 100. We obtained TB treatment notifications from Brazil’s National Notifiable Disease Information System (SINAN) (*5*) and death data from the Mortality Information System (SIM) (*6*), representing 438,163 notified TB cases and 45,984 TB-related deaths. Using these data, we estimated a Bayesian model of tuberculosis incidence (M.H. Chitwood et al., unpub. data, https://doi.org/10.2139/ssrn.3463278) in which incidence is approximated by the sum of 3 numbers: treatment initiations, deaths before treatment initiation, and disease resolutions before treatment initiation for a municipality in a given year. We reported the annual incidence rate as absolute incidence divided by population size and the fraction receiving treatment (fraction treated) as the number initiating treatment divided by incidence in a given year. The fraction treated differs from the case detection rate by considering loss to follow-up between diagnosis and treatment as an additional mechanism contributing to undertreatment. We also estimated the incidence of untreated TB (untreated TB rate) as the product (incidence rate) × (1 − fraction treated), to produce a combined measure of elevated incidence and inadequate case detection. 

**Figure 1:**
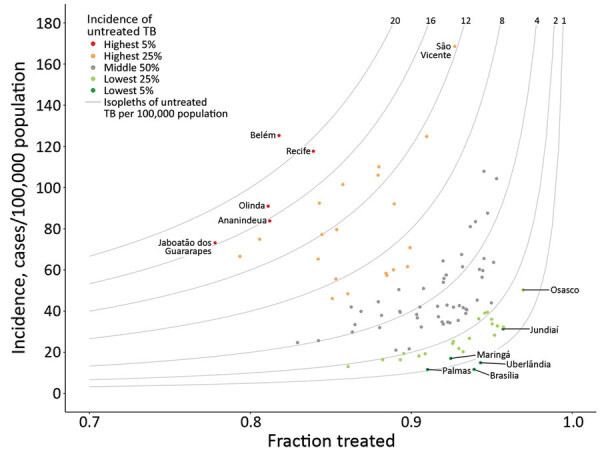
Modeled tuberculosis (TB) burden in 101 largest municipalities and state capitals of Brazil, 2017. Gray curves indicate isopleths of untreated TB incidence × (1 − fraction treated). Municipalities in the 5th and 95th percentiles of untreated TB, as well as those with the highest incidence (São Vicente) and highest fraction treated (Osasco), are labeled.

Across all 101 municipalities in 2017, there were 53.2 treatment notifications/100,000 population; we estimate a TB incidence rate of 58.6 (range 11.6–169) cases/100,000 population (Table). In 2017 São Vicente had the highest estimated TB incidence, 169 (95% CI 154–185) cases/100,000 population, and Palmas had the lowest, 11.6 (95% CI 9.3–14.3) cases/100,000 population. We estimate that the fraction treated ranged from 0.778 (95% CI 0.687–0.852) to 0.969 (95% CI 0.934–0.990)/100,000 population and the untreated TB rate ranged from 0.723 (95% CI 0.231–1.61) to 23.0 (95% CI 15.1–34.8)/100,000 population (Figure 1). 

**Table:** Reported cases and estimated burden of TB in state capitals of Brazil, 2017

Municipality	Case notifications/100,000 population*	Incidence/100,000 population (95% CI)	Fraction of cases treated (95% CI)	Untreated TB/100,000 population (95% CI)†
Rio Branco	82.7	83.5 (75.0–92.9)	0.940 (0.879–0.979)	5.07 (1.72–10.7)
Maceió	47.2	55.6 (50.5–61.6)	0.853 (0.779–0.908)	8.23 (4.80–13.4)
Manaus	114	125 (118–133)	0.910 (0.855–0.946)	11.4 (6.51–19.2)
Macapá	39.0	39.9 (34.7–46.3)	0.893 (0.798–0.956)	4.34 (1.64–8.92)
Salvador	54.6	65.3 (60.6–71.9)	0.842 (0.765–0.898)	10.4 (6.31–16.8)
Fortaleza	63.7	70.8 (66.7–75.8)	0.899 (0.849–0.938)	7.17 (4.25–11.3)
Vitória	36.3	39.4 (34.3–44.9)	0.947 (0.883–0.984)	2.10 (0.604–5.00)
Goiânia	17.1	19.4 (17.3–21.7)	0.895 (0.815–0.953)	2.05 (0.883–3.80)
São Luís	64.5	77.2 (70.8–85.0)	0.844 (0.771–0.902)	12.1 (7.20–19.1)
Belo Horizonte	23.6	25.3 (23.2–27.7)	0.926 (0.861–0.97)	1.88 (0.702–3.73)
Campo Grande	38.7	42.4 (38.3–47.2)	0.916 (0.847–0.964)	3.58 (1.45–6.98)
Cuiabá	68.6	79.6 (68.9–103)	0.854 (0.650–0.947)	12.2 (3.82–35.7)
Belém	103	125 (116–138)	0.818 (0.744–0.872)	23.0 (15.1–34.8)
João Pessoa	47.9	51.0 (45.8–57.1)	0.908 (0.824–0.962)	4.78 (1.83–9.80)
Recife	98.4	118 (110–129)	0.839 (0.770–0.892)	19.0 (12.1–29.5)
Teresina	27.6	32.3 (28.8–36.6)	0.906 (0.817–0.966)	3.07 (1.04–6.45)
Curitiba	17.0	19.3 (17.4–21.6)	0.909 (0.829–0.962)	1.78 (0.693–3.57)
Rio de Janeiro	99.8	104 (101–109)	0.953 (0.917–0.977)	4.93 (2.33–8.98)
Natal	54.0	58.3 (52.9–64.8)	0.884 (0.809–0.940)	6.81 (3.36–11.9)
Porto Velho	75.9	81 (73.6–89.5)	0.937 (0.869–0.978)	5.19 (1.71–11.2)
Boa Vista	44.0	41 (35.4–47.1)	0.934 (0.865–0.976)	2.73 (0.914–5.99)
Porto Alegre	92.9	106 (99.2–115)	0.879 (0.817–0.924)	12.9 (7.65–20.8)
Florianópolis	39.3	45.4 (40.5–51.1)	0.941 (0.868–0.983)	2.71 (0.752–6.51)
Aracaju	39.1	42 (37.6–47.3)	0.905 (0.829–0.960)	4.02 (1.59–7.77)
São Paulo	56.5	59.7 (57.5–62.5)	0.944 (0.904–0.972)	3.33 (1.6–6.04)
Palmas	6.28	11.6 (9.34–14.3)	0.910 (0.786–0.974)	1.06 (0.279–2.75)
Rio Branco	82.7	83.5 (75.0–92.9)	0.940 (0.879–0.979)	5.07 (1.72–10.7)

*Excluding notifications for misdiagnosis, reengagement in care, and deceased persons. †Untreated TB is the product of incidence × (1 − fraction treated), rounded up.

During 2008–2017, there were 438,163 TB treatment notifications; for this period we estimate that there were 488,329 (95% CI 474,715–507,676) incident TB cases, of which 49,778 (95% CI 36,072–69,217) did not initiate treatment. We observed a decrease in notifications from 56.6/100,000 population in 2008 to 53.2/100,000 population in 2017; over this period we estimate that average incidence decreased from 63.9 (range 13.7–138) to 58.6 (range 11.6–169)/100,000 population. Incidence decreased at an average annual rate of 0.95% (range −5.41% to 4.73%), the fraction treated increased at an average annual rate of 0.290% (range −0.966% to 3.55%), and the untreated TB rate decreased at an average annual rate of 2.88% (range −17.4% to 7.98%).

**Figure 2:**
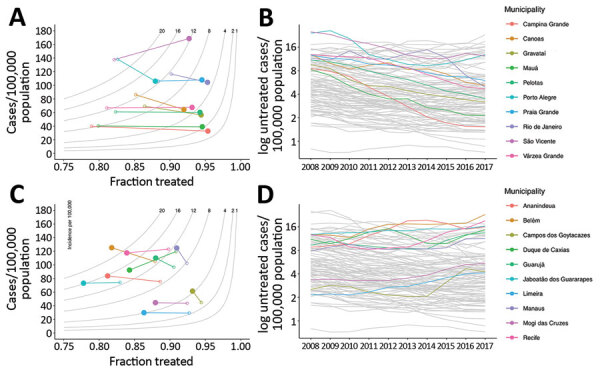
Municipalities of Brazil with the greatest decreases and increases in untreated tuberculosis (TB), 2008–2017. A, B) The 10 municipalities with the greatest decrease in untreated TB, showing the difference between modeled incidence and fraction treated (panel A) and time series of untreated TB (B). C) The 10 municipalities of Brazil with the greatest increase in untreated TB, showing the difference in modeled incidence of TB and fraction treated (C) and time series of untreated TB (D). In panels A and C, gray lines represent isopleths of untreated TB rate per 100,000 population, measured as the product of incidence and (1 − fraction treated); open circles indicate 2008 values, solid circles 2017 values. In panels B and D, gray lines represent other municipalities for comparison.

We compared the 10 municipalities with the largest absolute decrease and the 10 with the largest absolute increase in the untreated TB rate (Figure 2). In the municipalities with the largest decrease in untreated TB, the fraction of treated TB cases increased at an average annual rate of 1.23% (0.619–2.17), and incidence decreased at an average annual rate of 1.31% (−3.16 to 2.31) (Figure 2, panels A, B). We estimated that incidence increased in 2/10 municipalities, most notably São Vicente, which had an average annual rate of increase of 2.31% (95% CI 0.642%–3.89%).

In the 10 municipalities with the largest increase in untreated TB, the fraction treated decreased; average annual rate was 0.596% (0.252–0.985) and average incidence rate increased (0.732%; range −2.82 to 3.62) (Figure 2, panels C, D). Although the fraction treated decreased on average, CIs were wide and crossed 0 for the majority of estimates. The change in incidence was heterogenous in this group, ranging from an average decrease of 2.83% (95% CI 1.75%–3.93%) per year in Duque de Caxias to an average increase of 3.63% (95% CI 1.82%–5.35%) per year in Campos dos Goytacazes.

## Conclusions

Using a recently developed Bayesian approach for subnational TB estimation (M.H. Chitwood et al., unpub. data), we estimated the TB incidence rate, fraction treated, and the untreated TB rate for 101 large municipalities in Brazil during 2008–2017. We found that the incidence rate decreased on average and the fraction treated increased on average over the study period. However, in several high-burden municipalities, TB incidence rose and the fraction treated declined, increasing the untreated-TB rate and indicating gaps in local TB control efforts. Comparing our results with a similar state-level analysis of TB trends in Brazil, we found that large municipalities are more heterogenous and have more volatile trends in incidence and fraction treated than states.

The rate of untreated TB communicates both the size of the epidemic and the strength of the response. Municipalities with the highest incidence or the lowest fraction treated may not be the same municipalities with the highest untreated TB rate; an area with a moderate TB incidence and a moderate fraction treated could have a nontrivial rate of untreated TB. If municipalities in need of additional programmatic support were identified based only on the estimated incidence or fraction treated, cities with moderate incidence may be overlooked.

Because we applied a common set of assumptions across all municipalities, our approach may not account for local factors that influence the ratio between reported TB cases and deaths attributed to TB. In our analysis, this ratio provides a signal of the completeness of case detection. If TB death reporting in a municipality were biased downwards (e.g., many TB deaths were misattributed to other causes), the result would be an upward bias in the estimate of the fraction of cases treated. We assume that differences between deaths of persons who have initiated treatment and deaths reported in SIM are due to deaths that occur before treatment. A records linkage of SIM and SINAN was not possible for this analysis. Such a linkage would enable more precise quantification of the frequency of death before treatment initiation. If the overlap between the systems was lower than expected (e.g., more deaths before treatment initiation), our model would underestimate TB burden.

In this analysis, we identified municipalities, such as São Vicente, in which both the fraction treated and incidence increased on average. If these estimates are correct, our findings suggest that factors other than treatment coverage, such as delays between disease onset and treatment initiation, low treatment completion rates, or worsening nutrition and housing quality, could be driving trends in TB incidence. Further analysis of municipalities with both increasing fraction treated and increasing incidence is warranted to elucidate which factors drive increasing TB incidence despite improvements in treatment coverage.

Appendix Additional information about trends in untreated tuberculosis in large municipalities, Brazil, 2008–2017.
