# Supplementary material for: Trends in Untreated Tuberculosis in Large Municipalities, Brazil, 2008–2017
Source: Emerg Infect Dis. 2021 Mar;27(3):957–60. doi: 10.3201/eid2703.204094 (PMC7920690; doi:10.3201/eid2703.204094)
Supplement: Appendix — Additional information about trends in untreated tuberculosis in large municipalities, Brazil, 2008–2017. [file 20-4094-Techapp-s1.pdf]

Article DOI: https://doi.org/10.3201/eid2703.204094

Trends in Untreated Tuberculosis in Large Municipalities, Brazil, 2008–2017

Appendix

Data

We selected the 100 most populous municipalities in Brazil, based on the mean population over the study period (2008–2017), plus a state capital not included in the initial 100. We obtained TB case notifications from 2008–2017 from the National Notifiable Disease Information System (SINAN; Sistema de Informação de Agravos de Notificação) (*1*). We selected cases with reported residence in one of the sampled municipalities (n = 506,185). Cases were excluded if the individual was coded as misdiagnosed (n = 9,598), reentering treatment after being presumed lost to follow-up (n = 42,261), reentering treatment after transferring from another clinic (n = 15,077), or diagnosed postmortem (n = 1,666). We analyzed data for 438,163 notified TB cases.

We obtained mortality data from 2008–2017 from the national Mortality Information System (SIM; Sistema de Informação de Mortalidade) (*2*). We determined that a person had died with TB if their death record contained (as a primary or secondary cause) a code from the International Classification of Diseases, 10th edition, related to TB, including those related to TB-HIV (*3*) (n = 45,984). Deaths were included in our analysis if the person resided in one of the included municipalities immediately before their demise. We used established methods to account for underreporting of TB as a cause of death and incomplete mortality system coverage (*4*,*5*). Estimates of mortality system completeness were available only at the state level; we applied these estimates to municipalities in each state.

We used results from an expert opinion survey to estimate the fraction of persons recovering from active TB without treatment (M. Chitwood et al., unpub.data, https://doi.org/10.2139/ssrn.3463278). We assumed that this value is proportional to the fraction of TB-related deaths that occur among persons who die before receiving treatment.

Finally, we obtained municipality-level sociodemographic covariates describing wealth level and healthcare access, 2 factors thought to be associated with TB incidence and the fraction receiving treatment. We used GDP per capita to describe wealth level (*6*). As an indicator of healthcare access we used municipal-level Family Health Strategy coverage (*7*); the Family Health Strategy is Brazil's method of delivering primary care (*8*). Finally, we obtained municipal-level population estimates for each study year from the Brazilian Institute of Geography and Statistics (Instituto Brasileiro de Geografia e Estatistica, IBGE) (*6*).

All data were deidentified and extracted from publicly available sources.

Model Description

The model estimates incidence as the number of persons exiting a state of undetected and untreated TB. Persons can exit this state by treatment initiation, death (before treatment initiation), or recovery (before treatment initiation). We estimated TB incidence, the fraction of cases receiving treatment, and the number of untreated cases (Appendix Table 1) as well as the average annual rates of change in these measures over the study period (Appendix Table 2).

We specified Poisson likelihood functions for SINAN case notification data and SIM mortality data.

$$Treatment\ Notifications_{ij} \sim Poisson(\gamma_{ij} \cdot \alpha_{ij} \cdot \beta_{ij})$$

$$TB\ Mortality_{ij} \sim Poisson\left(\gamma_{ij} \cdot \alpha_{ij} \cdot \left[(\beta_{ij} * \delta_{ij}) + \left((1 - \beta_{ij}) \cdot (1-\mu)\right)\right] \cdot \pi_h \cdot \rho_{ij}\right)$$

For municipality *i* in year *j*, where γ_{ij} represents population size, α_{ij} represents the modeled TB incidence rate, β_{ij} represents the modeled fraction of treated cases, δ_{ij} represents the probability of death after treatment initiation, μ represents the probability of recovery without treatment, π_h represents the estimated coverage of SIM (calculated at the state-level, denoted *h*), and ρ_{ij} is an adjustment for misreporting of TB deaths in the SIM database, described below.

We specified exponential and inverse logit functions for incidence (α_{ij}) and fraction treated (β_{ij}), respectively:

$$\alpha_{ij} = exp(\varphi_0 + X_{ij}\varphi + \lambda_{ij})$$

$$\beta_{ij} = logit^{-1}(\omega_0 + X_{ij}\omega + \kappa_{ij})$$

For municipality i in year j, where φ_0 and ω_0 are intercepts, X_{ij} is a vector of the standardized municipal-level covariates (primary care access and log GDP per capita), and φ and ω are the associated vectors of regression coefficients for incidence and fraction treated respectively. The inclusion of these variables allows for partial pooling among municipalities with similar sociodemographic characteristics. Additionally, λ_{ij} and κ_{ij} are municipality-time random effects for incidence and fraction treated respectively. For each municipality these random effects are assumed to follow a random walk:

$$\lambda_{ij} = \psi_0 + \psi_{1,i} \cdot \sigma_{\psi 1} + \psi_{2,ij-1} \cdot \sigma_{\psi 2}$$

$$\kappa_{ij} = \phi_0 + \phi_{1,i} \cdot \sigma_{\phi 1} + \phi_{2,ij-1} \cdot \sigma_{\phi 2}$$

For municipality i in year j, where ψ_0 and ϕ_0 are intercepts, ψ_{1i} and ϕ_{1i} are demeaned municipal-level random effects; ψ_{2ij} and ϕ_{2ij} are demeaned autoregressive municipality-year effects, set equal to zero at $j = 1$; and $\sigma_{\psi 1}$, $\sigma_{\psi 2}$, $\sigma_{\phi 1}$, and $\sigma_{\phi 2}$ are standard deviation terms.

We estimated the probability of death among persons who initiated treatment (δ_{ij}):

$$\delta_{ij} = b_{ij} + c_{ij} \cdot \tau$$

Where b_{ij} is the probability that the treatment outcome is "death," c_{ij} is the probability that the treatment outcome is "loss to follow up," and τ is the probability that an individual dies given that they were lost to follow up. Values for b_{ij} and c_{ij} were estimated via logistic regression functions fitted to data for persons with a treatment outcome recorded (97.3% of all treated persons):

$$b_{ij} = logit^{-1}(X_{ij}\nu)$$

$$c_{ij} = logit^{-1}(X_{ij}\Omega)$$

For cases in municipality i in year j, where $X_{ij}\nu$ and $X_{ij}\Omega$ are vectors of covariates (including family health strategy coverage, GDP per capita (log scale), and a year fixed affect) with their associated regression coefficients. These regression estimates were used in preference to raw values to reduce the stochastic variation in the reported rates of these measures. We conducted a sensitivity analysis in which we coded all persons without a recorded treatment outcome as having abandoned treatment. While this led to an increase in the probability of abandonment, it did not meaningfully change estimates of incidence or fraction treated.

Finally, we estimated the systematic underreporting of TB as a cause of death (ρ_{ij}):

$$\rho_{ij} = 1 - \ logit^{-1}(\theta_0 + \ \eta_i \cdot \sigma_\eta + \theta_{j,1} \ \cdot (j - 10) + x_{ij}\theta_2)$$

For municipality *i* in year *j*, θ_0 is the intercept, η_i is a demeaned municipal-level random effect, σ_η is the random-effects variance, θ_1 is a linear time trend, x_{ij} is the percentage deaths in SIM attributed to a poorly-defined cause, and θ_2 is the associated regression coefficient.

There is substantial uncertainty around true values for several model parameters. We used a Bayesian approach to represent and propagate this uncertainty through the analysis. We used prior probability distributions to summarize existing evidence on all model parameters (Appendix Table 3). The prior probabilities of μ, and θ_3 were elicited through an expert opinion survey, described below. The prior distribution for τ was assumed to be Beta(4.3, 81), which corresponds to a lower bound of 0.01 and an upper bound of 0.1 (*9*). Prior distributions for all other model parameters were chosen to be weakly informative to allow the model to be fit with limited external influence while still excluding implausible values. Candidate models were assessed based on the validity of model assumptions and plausibility of results based on programmatic knowledge.

The model was implemented using Stan and the rStan package for R (*10,11*). We ran the model for 3,250 iterations on 4 chains, and retained the last 1,000 draws from each chain. This produced 4,000 samples from the posterior distributions of each quantity of interest. Point estimates were calculated as the mean of these samples, and confidence intervals were calculated as the 97.5 and 2.5 percentiles. Where averages are reported, they describe the central tendency across the sample of municipalities and were not weighted by population size.

Stan code for the model is available at github.com/mel-hc/bayesian_subnat_est.

Model Priors from Expert Opinion

Prior distributions for the probability of recovery without treatment and underreporting of TB as a cause of death were created based on an expert opinion survey described by Chitwood et al . To review, the prior distribution for recovery without treatment was created based on median values for lowest, highest, and best-guess estimates of respondents. Incorporating expert opinion into the death adjustment was slightly more complex. Respondents were asked to estimate the

rate of TB death misclassification among HIV negative persons in SIM in 2017 as it relates to the quality of cause of death reporting in a state or municipality, where the rate of ill-defined cases of death is an indicator for cause of death reporting quality. Two scenarios were presented: Scenario A, in which ≈1% of deaths an ill-defined cause, and Scenario B, where ≈15% of deaths had an ill-defined cause. Estimates were summarized as Beta distribution parameters. We incorporated these estimates into the death adjustment (ρ_{ij}) estimate as follows:

$$adjustment = \theta_0 + \frac{1}{n}\sum_{i=1}^{n} \eta_i \cdot \sigma_\eta$$

$$Scenario\ A = logit^{-1}(adjustment + \theta_2 \cdot y)$$

$$Scenario\ B = logit^{-1}(adjustment + \theta_2 \cdot z)$$

With the prior distributions:

Scenario A ~ Beta(52.97, 451.2)

Scenario B ~ Beta(97.83, 285.8)

y ~ Normal(0.01, 0.001)

z ~ Normal(0.15, 0.001)

The parameters θ_0, η_i, and θ_2 are used to estimate the overall death adjustment, described above.

Model Performance

The total model run time was ≈37 minutes. There were no divergent transitions or iterations that saturated the maximum tree depth of 12. Key parameter prior distributions and posterior means, confidence intervals, effective sample sizes, and R-hat values are presented in Appendix Table 3.

Sensitivity Analysis

We tested the sensitivity of results to parameter prior distributions Altering individual model priors had little impact on model diagnostics or run time; while we observed differences in posterior distributions between the main run and sensitivity runs (Appendix Table 4), we did

not observe meaningful changes to the distribution of our outcomes of interest (Appendix Figure 3). In addition to testing weaker priors, we ran the model with probability of survival without treatment fixed at 30% or 70%. While point estimates for several outcomes changed, they fell within the confidence intervals reported in the main analysis, and the relative burden of disease among modeled cities did not change.

Notes on Outliers

In 2 cities, Rio de Janeiro and São Vicente, model outcomes were indicative of biased case notification or TB-related death data. In Rio de Janeiro, we observed a decrease in TB-related deaths in SIM from 11.7/100,000 population in 2014 to 7.6 in 2017. We also observed an increase in treatment notification rates from 90.7 to 99.8 over that same period. In response, the model predicts an increase in fraction treated from 0.86 (range 0.80–0.91) in 2014 to 0.95 (range 0.92–0.98) in 2017. From the model results alone, we cannot discern whether there was a rapid improvement in fraction treated (and, consequently, a decrease in TB-related deaths) or whether there was a rapid deterioration in the quality of death records. In the latter case, the downward bias in TB death reporting would lead to an upward bias in the fraction treated estimate.

In São Vicente, we observed an increase in both TB notification rates (from 113 to 141) and deaths on treatment in SINAN (from 3.7 to 8.1 deaths/100,000) over the period 2013–2016. Over the same period, the model estimates only a slight increase in TB deaths, from 12.4 (8.7–18.1) to 14.1 (10.1–19.7) per 100,000. In response, the model predicts both a small improvement in fraction treated over the period, from 0.90 (95% CI 0.81–0.96) to 0.92 (95% CI 0.86–0.97) and a large increase in TB incidence from 130 (95% CI 118– 147) to 169 (95% CI 154–186) over the period 2013–2017. A stable rate of TB mortality despite a rapid increase in new case notifications may indicate a rapidly improving TB surveillance program. However, it may also indicate increased misattribution of TB as a cause of death during an accelerating epidemic, which would lead to an upward bias in fraction treated.

References

1. Ministério da Saúde. SIM––Mortality Information System dataset [in Portuguese] 2008 [cited 2021 Jan 13]. http://tabnet.datasus.gov.br/cgi/tabcgi.exe?sinannet/cnv/tubercMG.def

2. Ministério da Saúde. SIM——Mortality Information System dataset [in Portuguese] 2008 [cited 2021 Jan 13]. http://tabnet.datasus.gov.br/cgi/deftohtm.exe?sim/cnv/obt10br.def

3 World Health Organization. International statistical classification of diseases and related health problems, 10th revision. Geneva: The Organization; 2016.

4. Queiroz BL, Freire FHMA, Gonzaga MR, Lima EEC. Completeness of death-count coverage and adult mortality (45q15) for Brazilian states from 1980 to 2010 [in Portuguese]. Rev Bras Epidemiol. 2017;20(Suppl 01):21–33. PubMed https://doi.org/10.1590/1980-5497201700050003

5. Schmertmann CP, Gonzaga MR. Bayesian estimation of age-specific mortality and life expectancy for small areas with defective vital records. Demography. 2018;55:1363–88. PubMed https://doi.org/10.1007/s13524-018-0695-2

6. Instituto Brasileiro de Geografia e Estatística. Sistema IBGE de Recuperação Automática – SIDRA. 2018 [accessed August 2018]. http://www.sidra.ibge.gov.br

7. Ministério da Saúde. Portal da Saúde. Brasil. 2018 [accessed August 2018]. http://www2.datasus.gov.br/DATASUS/index.php?area=01

8. Andrade MV, Coelho AQ, Xavier Neto M, Carvalho LR, Atun R, Castro MC. Brazil's Family Health Strategy: factors associated with programme uptake and coverage expansion over 15 years (1998–2012). Health Policy Plan. 2018;33:368–80. PubMed https://doi.org/10.1093/heapol/czx189

9. Militão de Albuquerque MFP, Batista JDL, Ximenes RAA, Carvalho MA, Diniz GTN, Rodrigues LC. Risk factors associated with death in patients who initiate treatment for tuberculosis after two different follow-up periods. Rev Bras Epidemiol. 2009; 12:513–22. https://doi.org/10.1590/S1415-790X2009000400001

10. Stan Development Team. Stan modeling language users guide and reference manual. Version 2.18.0. 2018. [cited 2021 Jan 15]. http://mc-stan.org

11. Stan Development Team. RStan: the R interface to Stan. R package version 2.17.3. 2018 [cited 2021 Jan 15]. http://mc-stan.org

Appendix Table 1. Reported cases and estimated TB incidence in 101 municipalities Brazil, 2017

Municipality, state*	Case notifications/ 100,000 population†	Cases/100,000 population (95% CI)	Fraction of cases treated (95% CI)	Cases of untreated TB/100,000 population (95% CI)
Rio Branco, AC	82.7	83.5 (75.0–92.9)	0.94 (0.88–0.98)	5.07 (1.72–10.7)
Maceió, AL	47.2	55.6 (50.5–61.6)	0.85 (0.78–0.91)	8.23 (4.80–13.4)
Manaus, AM	114.1	124.8 (118.0–133.1)	0.91 (0.86–0.95)	11.36 (6.51–19.2)
Macapá, AP	39.0	39.9 (34.7–46.3)	0.89 (0.80–0.96)	4.34 (1.64–8.9)
Camaçari, BA	33.0	33.7 (28.8–39.5)	0.92 (0.82–0.98)	2.75 (0.71–6.8)
Feira de Santana, BA	35.4	36.7 (32.4–41.5)	0.90 (0.83–0.96)	3.57 (1.55–6.8)
Salvador, BA	54.6	65.3 (60.6–71.9)	0.84 (0.77–0.90)	10.38 (6.31–16.8)
Vitória da Conquista, BA	17.5	24.8 (20.7–29.4)	0.83 (0.70–0.92)	4.29 (1.73–8.3)
Caucaia, CE	51.3	57.4 (50.4–65.8)	0.88 (0.80–0.95)	6.68 (2.90–12.9)
Fortaleza, CE	63.7	70.8 (66.7–75.8)	0.90 (0.85–0.94)	7.17 (4.25–11.3)
Brasília, DF	10.6	11.7 (10.6–13.0)	0.94 (0.87–0.98)	0.72 (0.23–1.6)
Cariacica, ES	38.5	46.1 (40.2–53.0)	0.85 (0.75–0.92)	6.94 (3.34–12.3)
Serra, ES	31.0	37.9 (33.3–43.4)	0.88 (0.78–0.95)	4.55 (1.69–9.4)
Vila Velha, ES	37.2	42.0 (37.0–48.0)	0.86 (0.77–0.94)	5.84 (2.54–10.9)
Vitória, ES	36.3	39.4 (34.3–44.9)	0.95 (0.88–0.98)	2.10 (0.60–5.0)
Goiânia, GO	17.1	19.4 (17.3–21.7)	0.90 (0.82–0.95)	2.05 (0.88–3.8)
Anápolis, GO	10.4	13.1 (10.7–15.8)	0.86 (0.74–0.94)	1.85 (0.67–3.8)
São Luís, MA	64.5	77.2 (70.8–85.0)	0.84 (0.77–0.90)	12.09 (7.20–19.1)
Belo Horizonte, MG	23.6	25.3 (23.2–27.7)	0.93 (0.86–0.97)	1.88 (0.70–3.7)
Betim, MG	13.6	16.4 (13.8–19.6)	0.88 (0.75–0.96)	1.97 (0.55–4.6)
Contagem, MG	14.1	16.4 (13.9–19.2)	0.89 (0.78–0.97)	1.78 (0.54–3.9)
Governador Valadares, MG	26.7	33.5 (28.2–39.7)	0.87 (0.75–0.94)	4.58 (1.79–9.2)
Juiz de Fora, MG	46.1	50.5 (45.1–56.5)	0.89 (0.81–0.95)	5.49 (2.43–10.0)
Montes Claros, MG	23.4	24.3 (20.8–28.5)	0.93 (0.84–0.98)	1.83 (0.56–4.1)
Ribeirão das Neves, MG	19.5	25.7 (21.5–31.0)	0.84 (0.71–0.93)	4.12 (1.59–8.5)
Uberaba, MG	19.8	21.7 (18.1–25.8)	0.90 (0.80–0.96)	2.23 (0.77–4.8)
Uberlândia, MG	14.2	15.0 (12.9–17.2)	0.94 (0.88–0.98)	0.86 (0.29–1.9)
Campo Grande, MS	38.7	42.4 (38.3–47.2)	0.92 (0.85–0.96)	3.58 (1.45–7.0)
Cuiabá, MT	68.6	79.6 (68.9–102.9)	0.85 (0.65–0.95)	12.20 (3.82–35.7)
Várzea Grande, MT	63.9	67.5 (59.5–76.7)	0.93 (0.86–0.97)	4.69 (1.71–10.3)
Ananindeua, PA	67.0	83.8 (74.2–97.0)	0.81 (0.70–0.89)	15.97 (8.44–28.4)
Belém, PA	102.6	125.3 (116.0–138.1)	0.82 (0.74–0.87)	22.98 (15.05–34.8)
Santarém, PA	33.7	39.9 (34.1–46.6)	0.87 (0.77–0.94)	5.30 (2.18–10.3)
Campina Grande, PB	32.9	32.8 (28.7–37.5)	0.95 (0.90–0.98)	1.54 (0.52–3.4)
João Pessoa, PB	47.9	51.0 (45.8–57.1)	0.91 (0.82–0.96)	4.78 (1.83–9.8)
Caruaru, PE	56.2	57.6 (50.9–65.6)	0.92 (0.85–0.97)	4.55 (1.78–9.4)
Jaboatão dos Guararapes, PE	55.5	73.2 (65.4–82.6)	0.78 (0.69–0.85)	16.37 (10.00–25.5)
Olinda, PE	74.5	91.0 (81.1–103.3)	0.81 (0.72–0.88)	17.33 (10.11–28.1)
Paulista, PE	52.4	66.6 (58.1–76.9)	0.79 (0.69–0.88)	13.89 (7.56–23.4)
Petrolina, PE	28.0	32.0 (27.3–37.3)	0.88 (0.77–0.95)	3.87 (1.42–7.8)
Recife, PE	98.4	117.6 (109.6–128.5)	0.84 (0.77–0.89)	19.04 (12.14–29.5)
Teresina, PI	27.6	32.3 (28.8–36.6)	0.91 (0.82–0.97)	3.07 (1.04–6.5)
Cascavel, PR	15.6	18.2 (15.2–21.9)	0.90 (0.80–0.97)	1.76 (0.58–3.9)
Curitiba, PR	17.0	19.3 (17.4–21.6)	0.91 (0.83–0.96)	1.78 (0.69–3.6)
Foz do Iguaçu, PR	37.5	42.9 (36.9–50.1)	0.92 (0.83–0.97)	3.39 (1.06–8.0)
Londrina, PR	25.6	26.8 (23.5–30.7)	0.94 (0.87–0.98)	1.73 (0.56–3.8)
Maringá, PR	15.7	17.1 (14.3–20.2)	0.92 (0.83–0.98)	1.31 (0.38–3.2)
Ponta Grossa, PR	16.8	20.3 (17.1–23.9)	0.93 (0.85–0.98)	1.39 (0.39–3.3)
São José dos Pinhais, PR	17.2	21.9 (18.4–25.9)	0.93 (0.84–0.98)	1.56 (0.42–3.9)
Belford Roxo, RJ	59.7	74.9 (66.7–84.9)	0.81 (0.72–0.88)	14.67 (8.57–23.6)
Campos dos Goytacazes, RJ	57.7	61.5 (55.2–68.6)	0.93 (0.87–0.97)	4.24 (1.60–8.8)
Duque de Caxias, RJ	75.1	92.4 (84.5–102.8)	0.84 (0.76–0.91)	14.66 (8.20–24.6)
Niterói, RJ	47.5	54.0 (48.3–60.9)	0.92 (0.84–0.97)	4.38 (1.49–9.1)
Nova Iguaçu, RJ	81.4	92.1 (84.7–100.4)	0.89 (0.82–0.94)	10.26 (5.40–17.3)
Petrópolis, RJ	36.2	37.3 (32.1–43.2)	0.92 (0.83–0.97)	3.13 (1.03–6.6)
Rio de Janeiro, RJ	99.8	104.3 (100.6–109.1)	0.95 (0.92–0.98)	4.93 (2.33–9.0)
São Gonçalo, RJ	53.0	60.1 (54.6–66.2)	0.89 (0.82–0.94)	6.73 (3.36–11.9)
São João de Meriti, RJ	87.3	101.5 (92.0–112.8)	0.86 (0.78–0.92)	14.58 (7.85–24.1)
Volta Redonda, RJ	62.6	63.7 (56.2–72.0)	0.95 (0.89–0.98)	3.14 (1.00–7.2)
Mossoró, RN	35.5	34.7 (29.8–40.3)	0.92 (0.84–0.97)	2.71 (0.86–5.9)
Natal, RN	54.0	58.3 (52.9–64.8)	0.88 (0.81–0.94)	6.81 (3.36–11.9)
Porto Velho, RO	75.9	81.0 (73.6–89.5)	0.94 (0.87–0.98)	5.19 (1.71–11.2)

Municipality, state*	Case notifications/ 100,000 population†	Cases/100,000 population (95% CI)	Fraction of cases treated (95% CI)	Cases of untreated TB/100,000 population (95% CI)
Boa Vista, RR	44.0	41.0 (35.4–47.1)	0.93 (0.86–0.98)	2.73 (0.91–6.0)
Canoas, RS	58.2	64.4 (57.2–72.5)	0.92 (0.85–0.97)	5.21 (2.10–10.3)
Caxias do Sul, RS	41.8	41.7 (37.0–46.9)	0.93 (0.87–0.97)	2.84 (1.08–5.6)
Gravataí, RS	52.3	56.5 (49.8–64.1)	0.94 (0.88–0.98)	3.19 (1.00–7.3)
Pelotas, RS	54.6	60.3 (53.6–67.9)	0.94 (0.88–0.98)	3.51 (1.16–7.7)
Porto Alegre, RS	92.9	106.0 (99.2–114.5)	0.88 (0.82–0.92)	12.86 (7.65–20.8)
Santa Maria, RS	47.8	48.4 (41.6–56.3)	0.86 (0.77–0.93)	6.82 (3.27–12.3)
Blumenau, SC	34.1	33.8 (29.3–39.0)	0.95 (0.89–0.98)	1.70 (0.50–4.0)
Florianópolis, SC	39.3	45.4 (40.5–51.1)	0.94 (0.87–0.98)	2.71 (0.75–6.5)
Joinville, SC	37.3	40.7 (36.0–46.0)	0.93 (0.85–0.98)	2.70 (0.78–6.5)
Aracaju, SE	39.1	42.0 (37.6–47.3)	0.91 (0.83–0.96)	4.02 (1.59–7.8)
Bauru, SP	64.8	65.6 (58.4–73.2)	0.95 (0.89–0.98)	3.63 (1.31–7.8)
Campinas, SP	34.7	36.1 (32.9–39.7)	0.95 (0.90–0.98)	1.82 (0.58–4.0)
Carapicuíba, SP	58.2	61.6 (54.5–69.8)	0.90 (0.81–0.96)	6.38 (2.58–12.5)
Diadema, SP	43.6	44.0 (38.8–49.6)	0.95 (0.89–0.98)	2.23 (0.72–5.0)
Franca, SP	20.2	21.1 (17.6–25.1)	0.89 (0.79–0.96)	2.34 (0.83–5.0)
Guarujá, SP	97.3	110.1 (98.0–124.5)	0.88 (0.79–0.95)	13.41 (5.27–25.7)
Guarulhos, SP	38.9	42.7 (39.3–46.6)	0.93 (0.87–0.97)	3.02 (1.18–5.9)
Itaquaquecetuba, SP	37.2	39.5 (33.8–46.3)	0.89 (0.79–0.96)	4.29 (1.40–8.8)
Jundiaí, SP	33.9	31.4 (27.3–36.1)	0.96 (0.90–0.99)	1.36 (0.40–3.2)
Limeira, SP	24.9	29.9 (25.1–35.2)	0.86 (0.75–0.95)	4.14 (1.50–8.3)
Mauá, SP	37.7	39.1 (34.4–44.2)	0.95 (0.89–0.98)	2.14 (0.70–4.8)
Mogi das Cruzes, SP	38.9	44.5 (38.7–51.3)	0.88 (0.78–0.95)	5.44 (2.05–10.9)
Osasco, SP	50.7	50.3 (45.7–55.0)	0.97 (0.93–0.99)	1.55 (0.51–3.4)
Piracicaba, SP	51.1	55.9 (49.3–63.2)	0.92 (0.84–0.97)	4.50 (1.49–9.6)
Praia Grande, SP	103.2	107.9 (97.7–119.5)	0.95 (0.89–0.98)	5.98 (1.92–13.3)
Ribeirão Preto, SP	35.3	38.4 (34.1–43.1)	0.90 (0.83–0.96)	3.74 (1.43–7.1)
Santo André, SP	32.2	34.8 (31.2–38.8)	0.92 (0.85–0.97)	2.83 (1.15–5.5)
Santos, SP	83.5	87.6 (79.5–96.2)	0.95 (0.90–0.98)	4.64 (1.64–9.6)
São Bernardo do Campo, SP	32.4	32.3 (29.0–35.8)	0.96 (0.91–0.99)	1.39 (0.45–3.1)
São José do Rio Preto, SP	30.8	34.3 (30.0–39.3)	0.92 (0.84–0.97)	2.64 (0.87–5.6)
São José dos Campos, SP	28.6	28.4 (25.3–31.9)	0.95 (0.90–0.98)	1.38 (0.44–3.0)
São Paulo, SP	56.5	59.7 (57.5–62.5)	0.94 (0.90–0.97)	3.33 (1.60–6.0)
São Vicente, SP	160.4	168.6 (154.1–185.5)	0.93 (0.86–0.97)	12.45 (4.25–25.5)
Sorocaba, SP	32.3	35.5 (31.6–40.0)	0.93 (0.85–0.98)	2.54 (0.80–5.6)
Suzano, SP	38.9	41.9 (36.2–48.5)	0.93 (0.85–0.98)	3.13 (0.99–7.0)
Taubaté, SP	37.0	38.9 (33.8–44.6)	0.93 (0.86–0.98)	2.60 (0.87–5.6)
Palmas, TO	6.3	11.6 (9.30–14.3)	0.91 (0.79–0.97)	1.06 (0.28–2.7)
Aparecida de Goiânia, GO	36.5	36.3 (32.1–40.7)	0.94 (0.88–0.98)	2.12 (0.78–4.5)

*AC, Acre; AL, Alagoas; AM, Amazonas; AP, Amapá; BA, Bahia; CE, Ceará; DF, Distrito Federal; ES, Espírito Santo; GO, Goiás; MA, Maranhão; MG, Minas Gerais; MS, Mato Grosso do Sul; MT, Mato Grosso; PA, Pará; PB, Paraíba; PE, Pernambuco; PI, Piauí; PR, Paraná; RJ, Rio de Janeiro; RN, Rio Grande do Norte; RO, Rondônia; RR, Roraima; RS, Rio Grande do Sul; SC, Santa Catarina; SE, Sergipe; SP, São Paulo; TO, Tocantins.
†Excluding notifications for misdiagnosis, reengagement in care, and deceased persons.

Appendix Table 2. Modeled average annual percent change in TB burden in 100 municipalities in Brazil, 2008–2017*

Municipality	No. cases/100,000 population (95% CI)	Fraction of cases treated (95% CI)	No. cases with untreated TB/100,000 population (95% CI)
Rio Branco, AC	2.00 (−0.04 to 3.92)	1.22 (−0.027 to 3.11)	−8.0 (−17.8 to 3.15)
Maceió, AL	−2.84 (−4.03 to −1.64)	0.02 (−0.842 to 0.93)	−2.9 (−8.2 to 2.67)
Manaus, AM	2.22 (1.42 to 3.01)	−0.17 (−0.671 to 0.37)	4.7 (−1.6 to 12.65)
Macapá, AP	0.16 (−1.87 to 2.19)	−0.34 (−1.517 to 0.74)	4.0 (−7.2 to 16.36)
Camaçari, BA	−3.95 (−6.21 to −1.74)	−0.28 (−1.573 to 0.62)	−0.3 (−14.4 to 14.90)
Feira de Santana, BA	−1.99 (−3.71 to −0.26)	0.52 (−0.551 to 1.71)	−5.7 (−14.1 to 3.62)
Salvador, BA	−2.77 (−3.56 to −2.02)	−0.44 (−1.053 to 0.19)	0.1 (−4.2 to 4.98)
Vitória da Conquista, BA	−0.40 (−3.01 to 2.19)	0.50 (−1.554 to 2.80)	−2.4 (−12.2 to 8.27)
Caucaia, CE	−1.12 (−3.00 to 0.74)	0.47 (−0.759 to 1.87)	−4.0 (−12.5 to 5.51)
Fortaleza, CE	−2.08 (−2.86 to −1.34)	0.21 (−0.312 to 0.82)	−3.6 (−8.1 to 1.21)
Brasília, DF	−2.23 (−3.70 to −0.81)	−0.07 (−0.830 to 0.59)	−0.9 (−11.9 to 11.99)
Cariacica, ES	−1.94 (−3.81 to −0.01)	−0.13 (−1.389 to 1.16)	−1.0 (−9.1 to 7.64)
Serra, ES	−4.80 (−6.51 to −3.09)	−0.67 (−1.909 to 0.31)	2.3 (−9.0 to 14.14)
Vila Velha, ES	−1.59 (−3.43 to 0.31)	−0.56 (−1.801 to 0.57)	3.3 (−6.5 to 15.21)
Vitória, ES	−4.66 (−6.52 to −2.80)	−0.01 (−0.790 to 0.79)	−4.1 (−17.4 to 10.83)
Anápolis, GO	−0.50 (−3.31 to 2.57)	−0.21 (−1.987 to 1.61)	1.3 (−10.6 to 15.38)
Aparecida de Goiânia, GO	2.90 (0.56–5.14)	1.55 (0.300–3.35)	−9.3 (−18.4 to 0.38)
Goiânia, GO	−0.07 (−1.77 to 1.69)	−0.01 (−1.045 to 1.09)	0.3 (−9.0 to 10.76)
São Luís, MA	0.73 (−0.46 to 1.93)	0.14 (−0.741 to 1.10)	0.1 (−5.3 to 5.72)
Belo Horizonte, MG	−3.87 (−5.03 to −2.75)	0.33 (−0.459 to 1.30)	−6.9 (−15.5 to 2.75)
Betim, MG	−5.41 (−7.72 to −2.94)	−0.48 (−2.246 to 0.81)	−1.2 (−14.8 to 13.29)
Contagem, MG	−4.58 (−6.74 to −2.43)	−0.26 (−1.655 to 0.98)	−2.1 (−14.6 to 11.35)
Governador Valadares, MG	−4.71 (−6.90 to −2.52)	−0.04 (−1.618 to 1.41)	−4.4 (−13.9 to 6.41)
Juiz de Fora, MG	1.18 (−0.62 to 2.89)	0.06 (−0.969 to 1.19)	1.0 (−8.1 to 10.92)
Montes Claros, MG	−1.44 (−3.78 to 0.99)	0.38 (−0.719 to 1.74)	−5.2 (−17.1 to 7.73)
Ribeirão das Neves, MG	−4.16 (−6.44 to −1.82)	0.09 (−1.697 to 1.80)	−4.7 (−13.9 to 5.36)
Uberaba, MG	−0.88 (−3.46 to 1.79)	0.39 (−1.053 to 2.07)	−3.6 (−15.1 to 8.80)
Uberlândia, MG	0.61 (−2.15 to 3.32)	3.55 (1.567–6.17)	−17.1 (−26.1 to −7.93)
Campo Grande, MS	−0.43 (−2.02 to 1.15)	0.40 (−0.490 to 1.47)	−3.9 (−12.6 to 5.29)
Cuiabá, MT	−0.65 (−2.26 to 0.91)	0.31 (−0.888 to 1.76)	−2.2 (−9.6 to 6.28)
Várzea Grande, MT	0.15 (−2.05 to 2.28)	1.58 (0.251–3.45)	−10.8 (−19.9 to −1.06)
Ananindeua, PA	1.19 (−0.44 to 2.87)	−0.97 (−2.198 to 0.32)	7.8 (−1.3 to 18.05)
Belém, PA	1.97 (1.05–2.94)	−0.80 (−1.553 to −0.07)	7.1 (1.7–13.11)
Santarém, PA	−3.15 (−5.32 to −0.97)	0.23 (−1.264 to 1.75)	−4.4 (−13.6 to 5.62)
Campina Grande, PB	−2.08 (−4.57 to 0.28)	2.17 (0.500–4.57)	−17.4 (−26.9 to −7.13)
João Pessoa, PB	−1.37 (−2.84 to 0.09)	−0.30 (−1.214 to 0.52)	2.5 (−7.8 to 14.17)
Caruaru, PE	4.73 (2.31–7.16)	1.80 (0.363–3.81)	−6.5 (−15.4– 2.62)
Jaboatão dos Guararapes, PE	−0.16 (−1.60 to 1.21)	−0.71 (−1.826 to 0.40)	3.0 (−2.5 to 8.62)
Olinda, PE	0.22 (−1.27 to 1.80)	0.05 (−1.125 to 1.34)	0.2 (−5.6 to 6.35)
Paulista, PE	−1.30 (−3.14 to 0.54)	−0.11 (−1.554 to 1.32)	−0.8 (−7.2 to 5.98)
Petrolina, PE	−0.76 (−3.07 to 1.65)	0.21 (−1.320 to 1.86)	−2.0 (−13.0 to 9.86)
Recife, PE	−0.50 (−1.30 to 0.32)	−0.75 (−1.394 to −0.13)	5.0 (−0.2 to 11.44)
Teresina, PI	−2.84 (−4.51 to −1.22)	0.42 (−0.693 to 1.66)	−6.1 (−15.6 to 3.95)
Cascavel, PR	−3.79 (−6.33 to −1.27)	−0.06 (−1.318 to 1.16)	−3.2 (−14.5 to 9.29)
Curitiba, PR	−3.77 (−5.14 to −2.42)	−0.11 (−1.006 to 0.78)	−2.3 (−11.7 to 8.78)
Foz do Iguaçu, PR	−2.23 (−4.27 to −0.09)	0.37 (−0.789 to 1.69)	−5.9 (−17.2 to 6.06)
Londrina, PR	0.08 (−2.00 to 2.16)	0.35 (−0.610 to 1.60)	−3.6 (−15.5 to 9.80)
Maringá, PR	−3.95 (−6.38 to −1.50)	−0.08 (−1.294 to 0.95)	−3.0 (−15.8 to 11.69)
Ponta Grossa, PR	−3.81 (−6.10 to −1.49)	0.19 (−0.898 to 1.29)	−6.2 (−18.6 to 7.15)
São José dos Pinhais, PR	−4.48 (−6.83 to −2.05)	0.06 (−1.121 to 1.26)	−5.0 (−18.6 to 9.77)
Belford Roxo, RJ	−2.00 (−3.42 to −0.58)	−0.46 (−1.553 to 0.68)	0.3 (−5.6 to 6.43)
Campos dos Goytacazes, RJ	3.63 (1.82–5.35)	−0.15 (−0.918 to 0.75)	6.9 (−5.9 to 21.73)
Duque de Caxias, RJ	−2.83 (−3.93 to −1.75)	−0.83 (−1.736 to −0.05)	3.7 (−2.9 to 11.42)
Niterói, RJ	−3.62 (−5.11 to −2.06)	−0.07 (−0.967 to 0.81)	−2.6 (−13.6 to 9.56)
Nova Iguaçu, RJ	−0.28 (−1.40 to 0.85)	0.22 (−0.524 to 1.00)	−1.8 (−7.8 to 4.35)
Petrópolis, RJ	−0.95 (−3.09 to 1.32)	0.34 (−0.866 to 1.73)	−3.9 (−15.6 to 8.67)
Rio de Janeiro, RJ	−1.21 (−1.79 to −0.72)	0.62 (0.240–1.16)	−9.1 (−13.4 to −4.80)
São Gonçalo, RJ	−1.14 (−2.37 to 0.09)	0.20 (−0.627 to 1.12)	−2.4 (−9.0 to 4.85)
São João de Meriti, RJ	−0.89 (−2.25 to 0.50)	−0.05 (−1.019 to 0.90)	−0.4 (−6.7 to 6.66)
Volta Redonda, RJ	3.58 (1.40–5.78)	0.49 (−0.379 to 1.89)	−2.6 (−15.6 to 11.95)
Mossoró, RN	−0.74 (−3.10 to 1.61)	0.30 (−0.832 to 1.64)	−3.4 (−15.7 to 10.03)
Natal, RN	−0.95 (−2.29 to 0.40)	−0.24 (−1.126 to 0.68)	1.4 (−6.6 to 10.28)
Porto Velho, RO	0.40 (−1.29 to 2.02)	0.86 (−0.157 to 2.35)	−7.4 (−17.5 to 3.39)
Boa Vista, RR	0.91 (−1.37 to 3.29)	0.29 (−0.703 to 1.52)	−2.1 (−14.0 to 11.29)
Canoas, RS	−3.16 (−4.89 to −1.45)	0.87 (−0.132 to 2.12)	−9.7 (−18.0 to −1.26)
Caxias do Sul, RS	2.52 (0.46–4.59)	1.00 (−0.111 to 2.40)	−6.0 (−15.9 to 4.40)

Municipality	No. cases/100,000 population (95% CI)	Fraction of cases treated (95% CI)	No. cases with untreated TB/100,000 population (95% CI)
Gravataí, RS	−2.24 (−4.15 to −0.27)	1.00 (−0.100 to 2.41)	−11.7 (−21.8 to −0.43)
Pelotas, RS	−0.10 (−2.14 to 1.89)	1.54 (0.329–3.31)	−12.1 (−21.3 to −2.79)
Porto Alegre, RS	−2.89 (−3.71 to −2.09)	0.70 (0.069–1.42)	−6.8 (−10.7 to −2.93)
Santa Maria, RS	−1.11 (−3.34 to 1.14)	1.15 (−0.430 to 3.14)	−6.0 (−13.9 to 2.04)
Blumenau, SC	1.53 (−0.98 to 4.05)	0.71 (−0.399 to 2.44)	−6.4 (−19.7 to 8.53)
Florianópolis, SC	−1.62 (−3.40 to 0.12)	0.40 (−0.545 to 1.63)	−6.3 (−18.3 to 6.58)
Joinville, SC	0.01 (−1.84 to 1.89)	0.13 (−0.869 to 1.21)	−1.5 (−14.3 to 12.69)
Aracaju, SE	0.78 (−0.90 to 2.54)	−0.31 (−1.229 to 0.55)	4.8 (−5.7 to 16.88)
Bauru, SP	2.84 (0.76–4.85)	1.08 (0.050–2.69)	−7.3 (−17.0 to 3.15)
Campinas, SP	−0.79 (−2.38 to 0.68)	0.91 (−0.042 to 2.23)	−10.3 (−20.7 to 0.69)
Carapicuíba, SP	2.31 (0.29–4.31)	0.84 (−0.335 to 2.58)	−2.9 (−11.4 to 6.30)
Diadema, SP	−0.58 (−2.63 to 1.47)	1.02 (−0.130 to 2.73)	−10.5 (−22.1 to 2.49)
Franca, SP	1.44 (−1.30 to 4.25)	0.30 (−1.253 to 2.17)	−0.2 (−12.1 to 13.07)
Guarujá, SP	1.46 (−0.19 to 3.06)	−0.32 (−1.357 to 0.63)	4.5 (−4.6 to 15.04)
Guarulhos, SP	−0.06 (−1.35 to 1.24)	0.73 (−0.040 to 1.69)	−6.8 (−14.4 to 0.82)
Itaquaquecetuba, SP	−0.73 (−2.91 to 1.47)	0.26 (−1.043 to 1.73)	−2.4 (−12.8 to 9.05)
Jundiaí, SP	−1.62 (−3.71 to 0.49)	0.36 (−0.390 to 1.46)	−7.0 (−19.5 to 6.91)
Limeira, SP	0.21 (−2.25 to 2.63)	−0.80 (−2.352 to 0.52)	8.0 (−5.7 to 22.67)
Mauá, SP	−0.32 (−2.52 to 1.79)	1.93 (0.467–3.88)	−14.2 (−23.9 to −4.08)
Mogi das Cruzes, SP	0.24 (−1.72 to 2.20)	−0.56 (−1.775 to 0.47)	6.1 (−5.2 to 18.63)
Osasco, SP	−0.14 (−1.78 to 1.45)	1.01 (0.135–2.32)	−13.7 (−23.8 to −2.67)
Piracicaba, SP	3.35 (1.37–5.37)	−0.16 (−1.143 to 0.78)	5.8 (−7.0 to 19.95)
Praia Grande, SP	0.19 (−1.50 to 1.82)	0.77 (−0.092 to 2.02)	−7.9 (−17.2 to 2.10)
Ribeirão Preto, SP	−0.47 (−2.15 to 1.22)	−0.06 (−1.077 to 0.96)	0.4 (−9.9 to 11.39)
Santo André, SP	−0.28 (−1.93 to 1.44)	0.30 (−0.604 to 1.33)	−3.0 (−12.1 to 7.30)
Santos, SP	−1.16 (−2.57 to 0.23)	0.40 (−0.273 to 1.25)	−6.5 (−16.1 to 3.56)
São Bernardo do Campo, SP	1.32 (−0.61 to 3.20)	0.89 (−0.079 to 2.36)	−9.1 (−19.9 to 2.98)
São José do Rio Preto, SP	−1.31 (−3.24 to 0.72)	0.33 (−0.724 to 1.53)	−4.6 (−15.3 to 7.30)
São José dos Campos, SP	−0.47 (−2.36 to 1.33)	0.40 (−0.361 to 1.43)	−5.9 (−17.4 to 6.58)
São Paulo, SP	−0.83 (−1.39 to −0.34)	0.39 (0.002–0.90)	−5.7 (−10.4 to −0.49)
São Vicente, SP	2.31 (0.64–3.89)	1.38 (0.319–2.91)	−7.6 (−14.7 to −0.06)
Sorocaba, SP	−1.18 (−2.99 to 0.62)	0.47 (−0.524 to 1.69)	−5.7 (−16.5 to 6.14)
Suzano, SP	1.38 (−0.98 to 3.80)	0.44 (−0.641 to 1.88)	−2.8 (−14.4 to 9.69)
Taubaté, SP	0.80 (−1.50 to 3.07)	0.52 (−0.605 to 1.99)	−4.3 (−16.2 to 9.29)
Palmas, TO	−5.09 (−8.10 to −2.03)	−0.08 (−1.690 to 1.24)	−4.5 (−17.8 to 10.75)

*Negative numbers indicate decreases in the metric. AC, Acre; AL, Alagoas; AM, Amazonas; AP, Amapá; BA, Bahia; CE, Ceará; DF, Distrito Federal; ES, Espírito Santo; GO, Goiás; MA, Maranhão; MG, Minas Gerais; MS, Mato Grosso do Sul; MT, Mato Grosso; PA, Pará; PB, Paraíba; PE, Pernambuco; PI, Piauí; PR, Paraná; RJ, Rio de Janeiro; RN, Rio Grande do Norte; RO, Rondônia; RR, Roraima; RS, Rio Grande do Sul; SC, Santa Catarina; SE, Sergipe; SP, São Paulo; TO, Tocantins.

Appendix Table 3. Prior and posterior distributions for key model parameters in study of TB incidence in Brazil, 2008–2017

		Posterior distribution				
Parameter	Prior distribution	Mean	Lower bound, 2.5%	Upper bound, 97.5%	Effective sample size	R-hat
φ_0	Normal(0, 10)	3.8811	3.8029	3.9612	793.5541	1.0024
φ_{GDP}	Normal(0, 10)	−0.0649	−0.1094	−0.0225	981.5023	1.0064
φ_{FHS}	Normal(0, 10)	−0.0024	−0.0309	0.0266	1154.3115	1.0015
$\sigma_{\varphi i0}$	Cauchy(0, 2)*	0.5345	0.4645	0.6154	4862.4026	0.9995
$\sigma_{\varphi ij}$	Cauchy(0, 2)*	0.0766	0.0690	0.0844	771.8664	1.0059
ω_0	Normal(0, 10)	2.0898	1.7013	2.4626	1206.1054	0.9999
ω_{FHS}	Normal(0, 10)	0.1064	−0.0079	0.2142	2598.8251	1.0018
ω_{GDP}	Normal(0, 10)	0.3874	0.2427	0.5335	2076.6624	1.0000
$\sigma_{\omega i0}$	Cauchy(0, 2)*	0.3459	0.2119	0.4993	686.5157	1.0020
$\sigma_{\omega ij}$	Cauchy(0, 2)*	0.2506	0.2050	0.2994	1754.2945	1.0013
θ_0	Normal(0, 1)	−1.6386	−1.8982	−1.3841	3052.2449	0.9994
θ_2	Normal(0, 0.05)	−0.0416	−0.0775	−0.0036	1798.5758	1.0009
θ_3	Normal(0, 1)	2.3960	0.9038	3.8694	7953.9136	0.9997
$\sigma_{\rho i0}$	Cauchy(0, 2)*	0.8070	0.5819	1.0706	1210.9874	1.0000
μ	Beta(25.65, 33.32)	0.5196	0.3776	0.6537	775.3464	1.0003
τ	Beta(4.29, 81.47)	0.0315	0.0092	0.0659	4949.3005	1.0000

*Cauchy distributions were implemented as half-cauchy (constrained to >0).

Appendix Table 4. Prior and posterior distributions for parameters modified during sensitivity analysis

		Posterior				
Parameter	Prior distribution	Mean	Lower bound, 2.5%	Upper bound, 97.5%	Effective sample size	R.hat
θ_2	Normal(0, 0.05)	−0.0416	−0.0775	−0.0036	1798.6	1.001
SA: θ_2	Normal(0, 0.1)	−0.0463	−0.0826	−0.006	1725.9	1.000
μ	Beta(25.65, 33.32)	0.5196	0.3776	0.6537	775.3	1.000
SA_1: μ	Beta(47.3, 47.3)	0.5575	0.4437	0.5974	651.7	1.003
SA_2: μ	Normal(0.3, 0.001)	0.3000	0.2980	0.3020	7663.4	0.999
SA_3: μ	Normal(0.7, 0.001)	0.7000	0.6981	0.7020	12553.9	0.999
τ	Beta(4.29, 81.47)	0.0315	0.0092	0.0659	4949.3	1.000
SA: τ	Beta(4.37, 50.25)	0.0415	0.013	0.085	4158.0	0.999
Scenario A	Beta(52.97, 451.2)	0.1647	0.1385	0.1918	4177.0	0.999
SA: Scenario A*	Beta(35.6, 321)	0.166	0.134	0.20	3602.0	1.001
Scenario B	Beta(97.83, 285.8)	0.2161	0.1828	0.2516	4791.1	1.000
SA: Scenario B*	Beta(40.7, 122)	0.198	0.156	0.246	4623.0	1.000

*Because they relate to the same parameter (θ_3), priors for Scenario A and Scenario B were altered together in the sensitivity analysis; all others were altered in isolation. SA, sensitivity analysis.

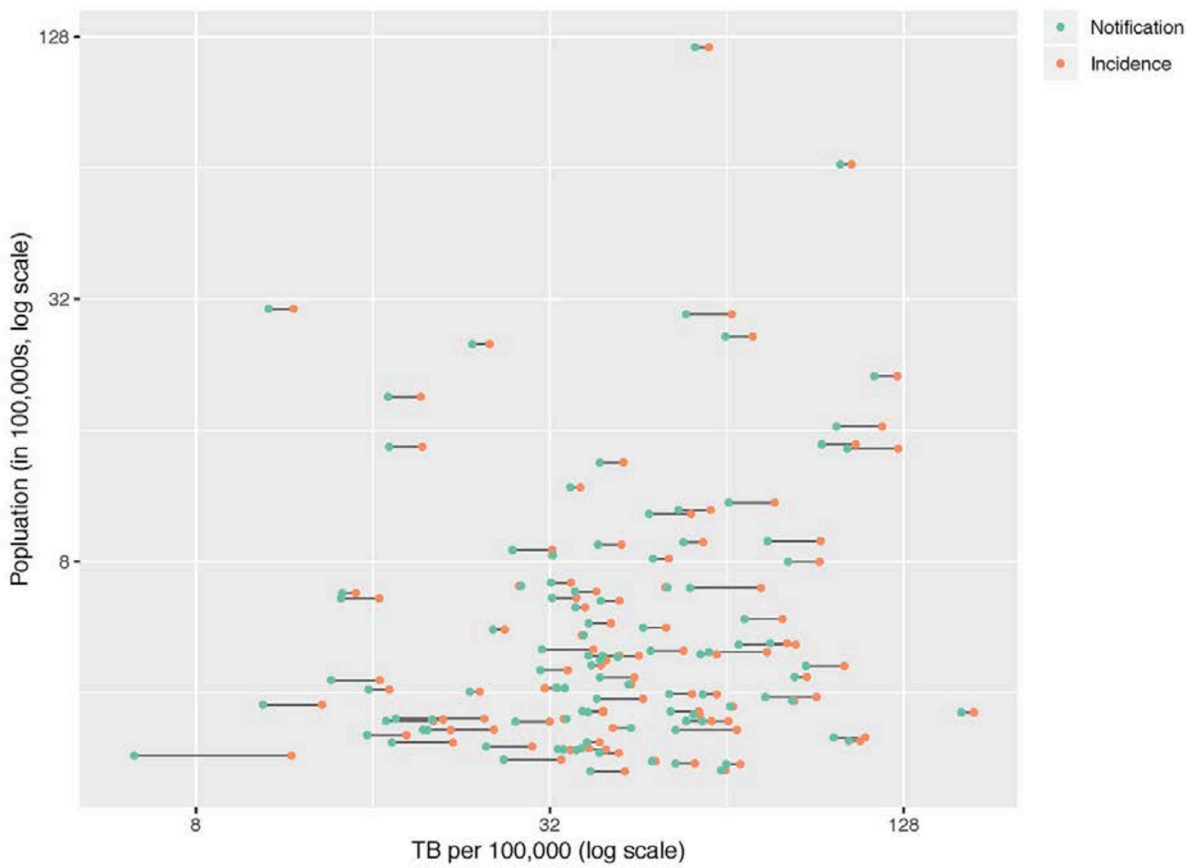

Appendix Figure 1. TB treatment notification rates and estimated incidence rates in 101 municipalities, Brazil, 2008–2017. Rates are apparently uncorrelated with population size.

Figure S2: Trends in Observed and Modeled TB Burden by Municipality, 2008 – 2017

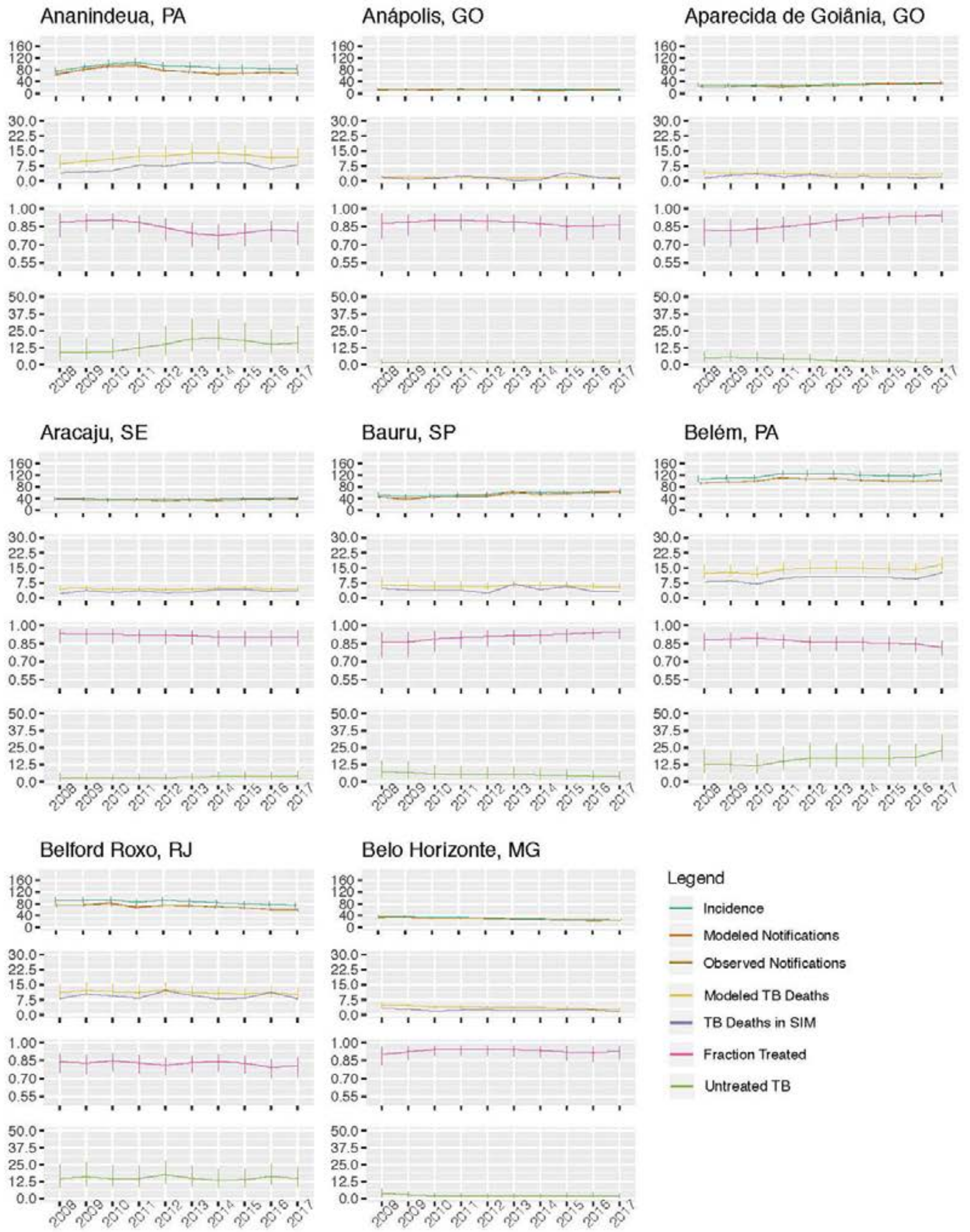

Figure S2: Trends in Observed and Modeled TB Burden by Municipality, 2008 – 2017

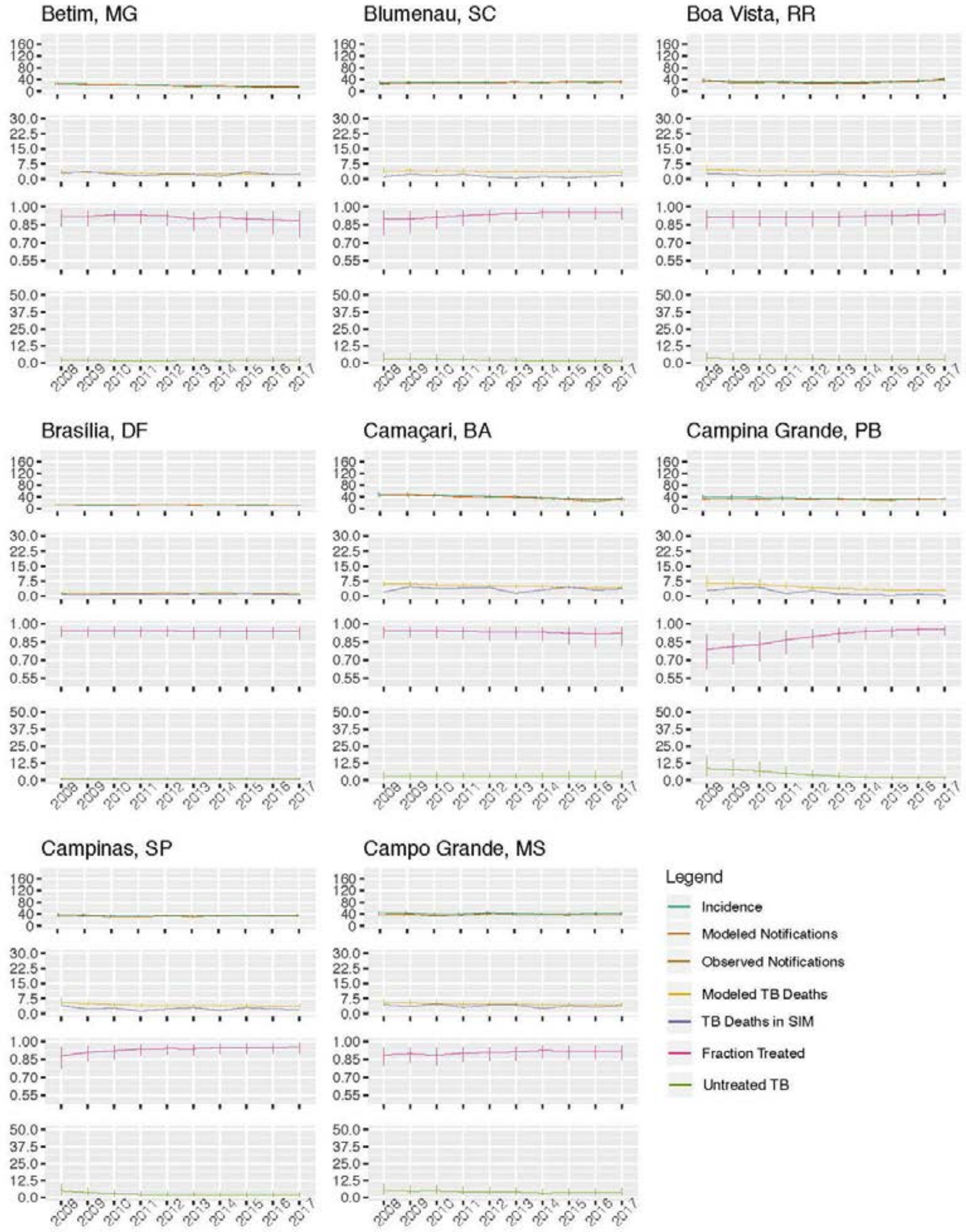

Figure S2: Trends in Observed and Modeled TB Burden by Municipality, 2008 – 2017

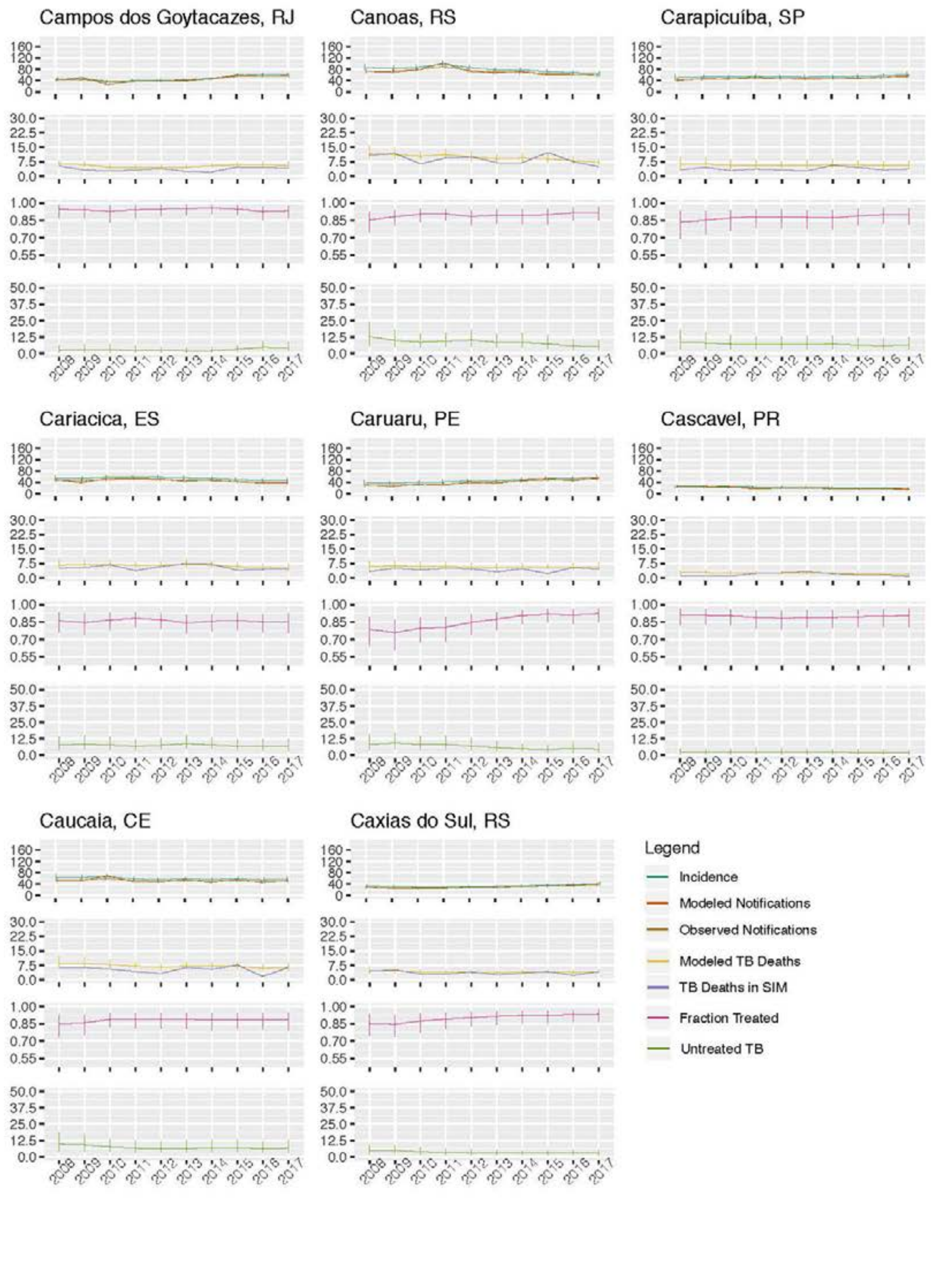

Figure S2: Trends in Observed and Modeled TB Burden by Municipality, 2008 – 2017

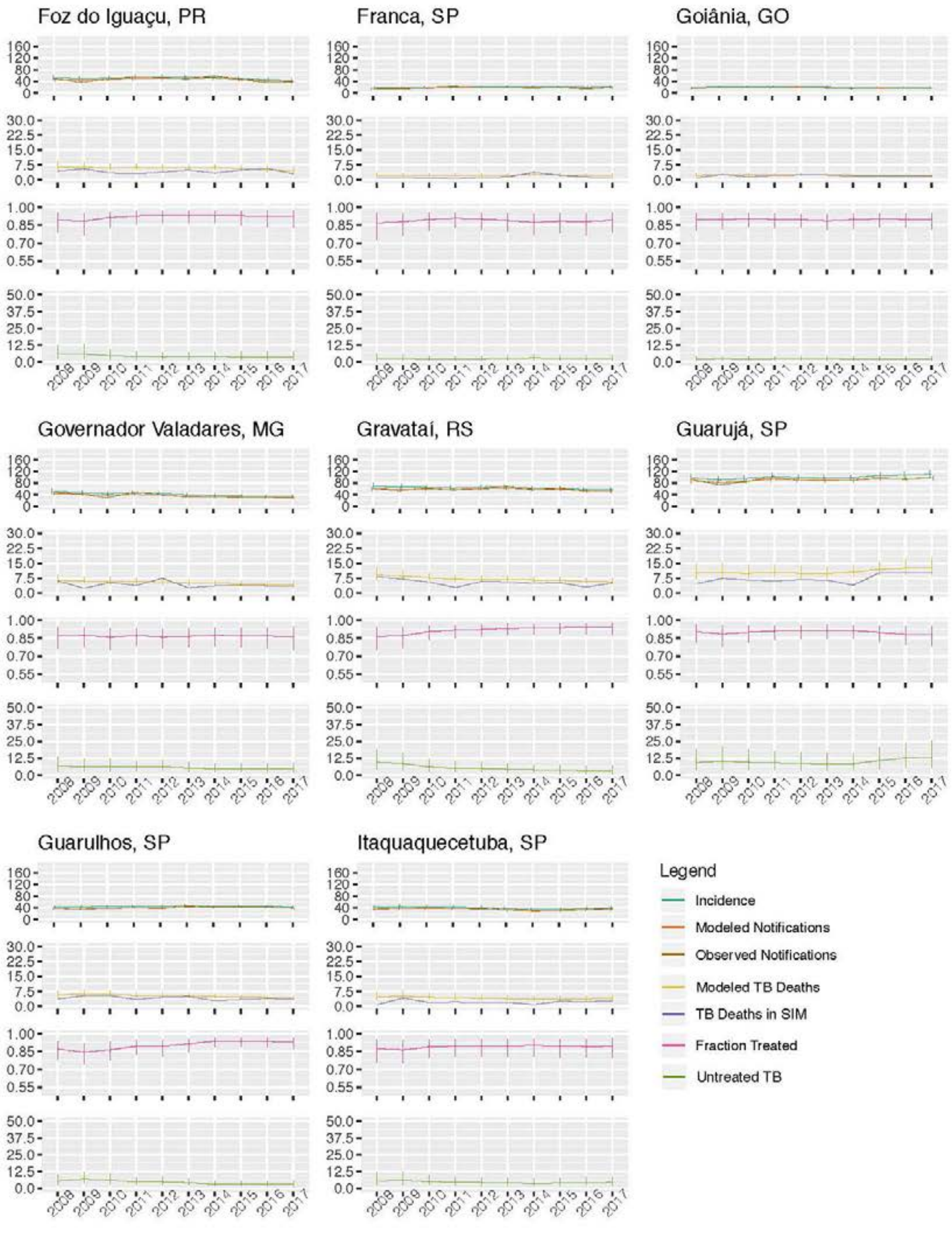

Figure S2: Trends in Observed and Modeled TB Burden by Municipality, 2008 – 2017

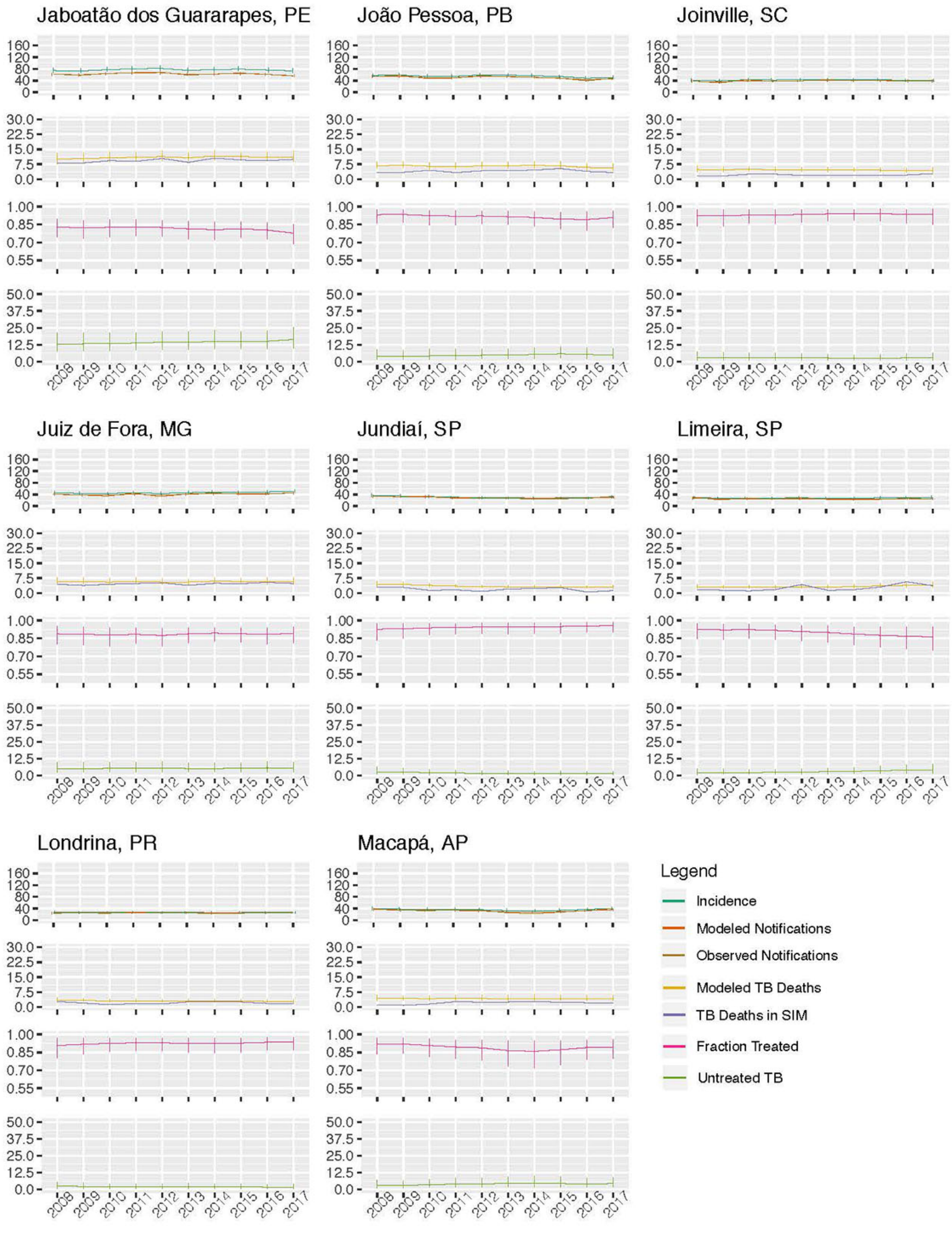

Figure S2: Trends in Observed and Modeled TB Burden by Municipality, 2008 – 2017

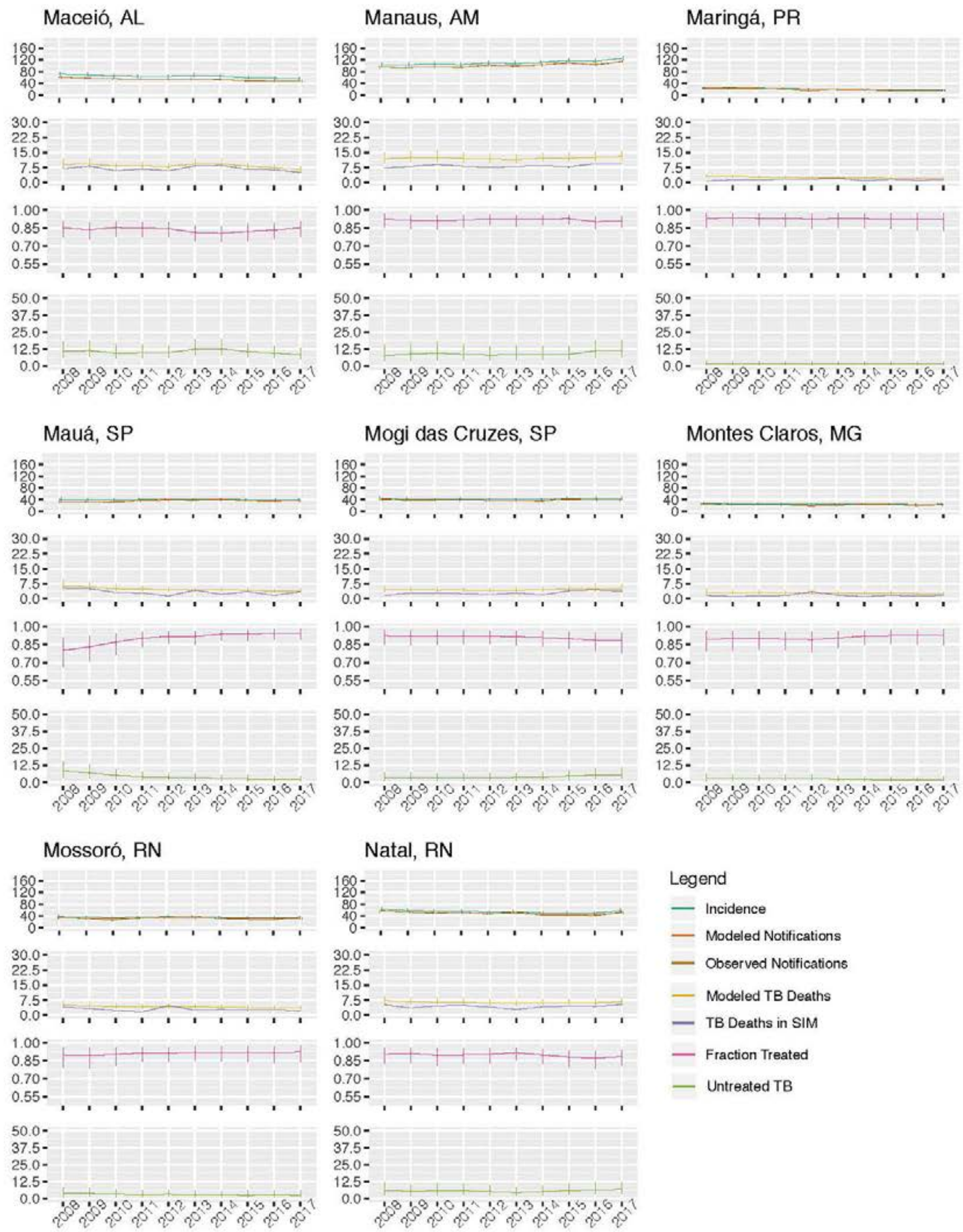

Figure S2: Trends in Observed and Modeled TB Burden by Municipality, 2008 – 2017

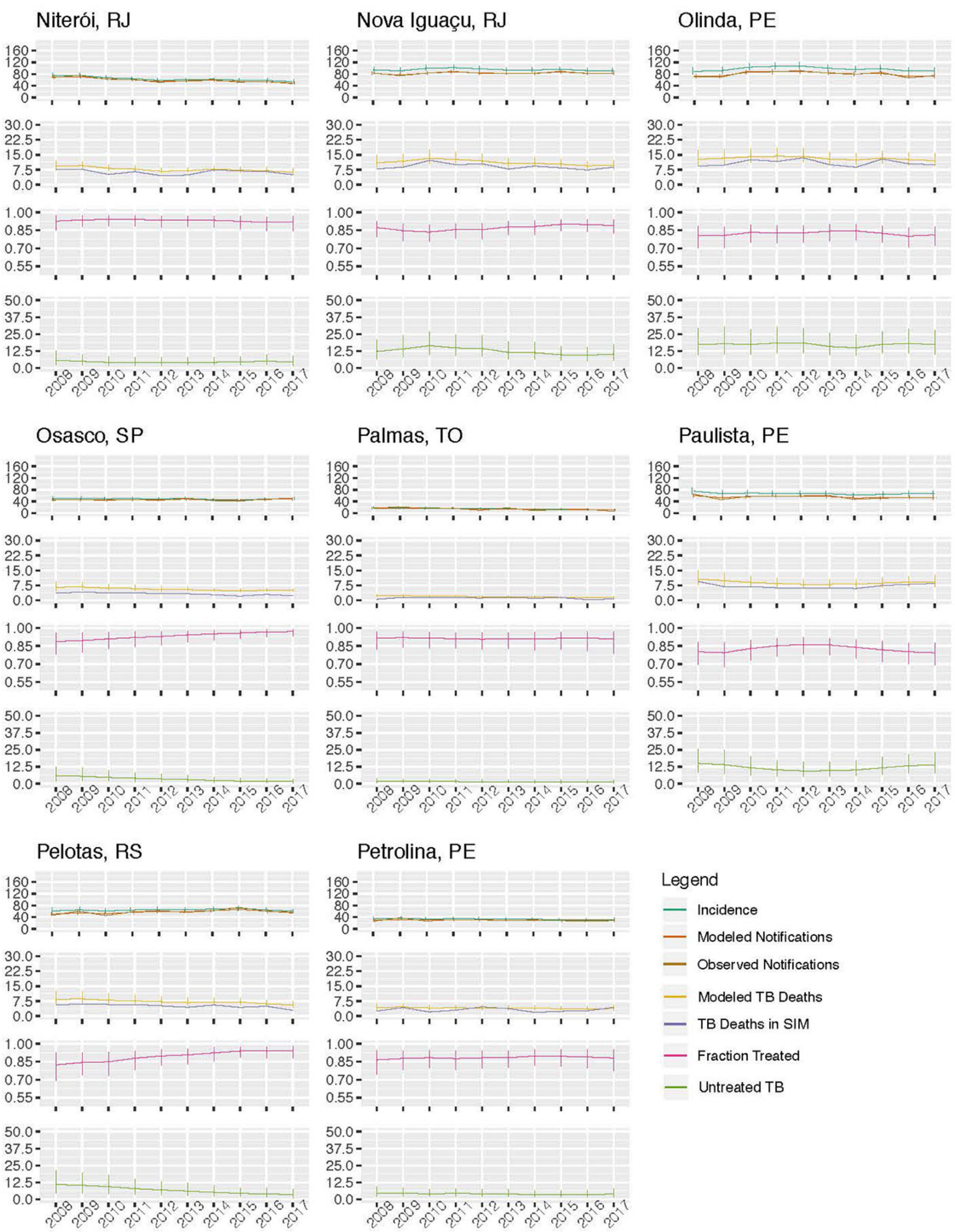

Figure S2: Trends in Observed and Modeled TB Burden by Municipality, 2008 – 2017

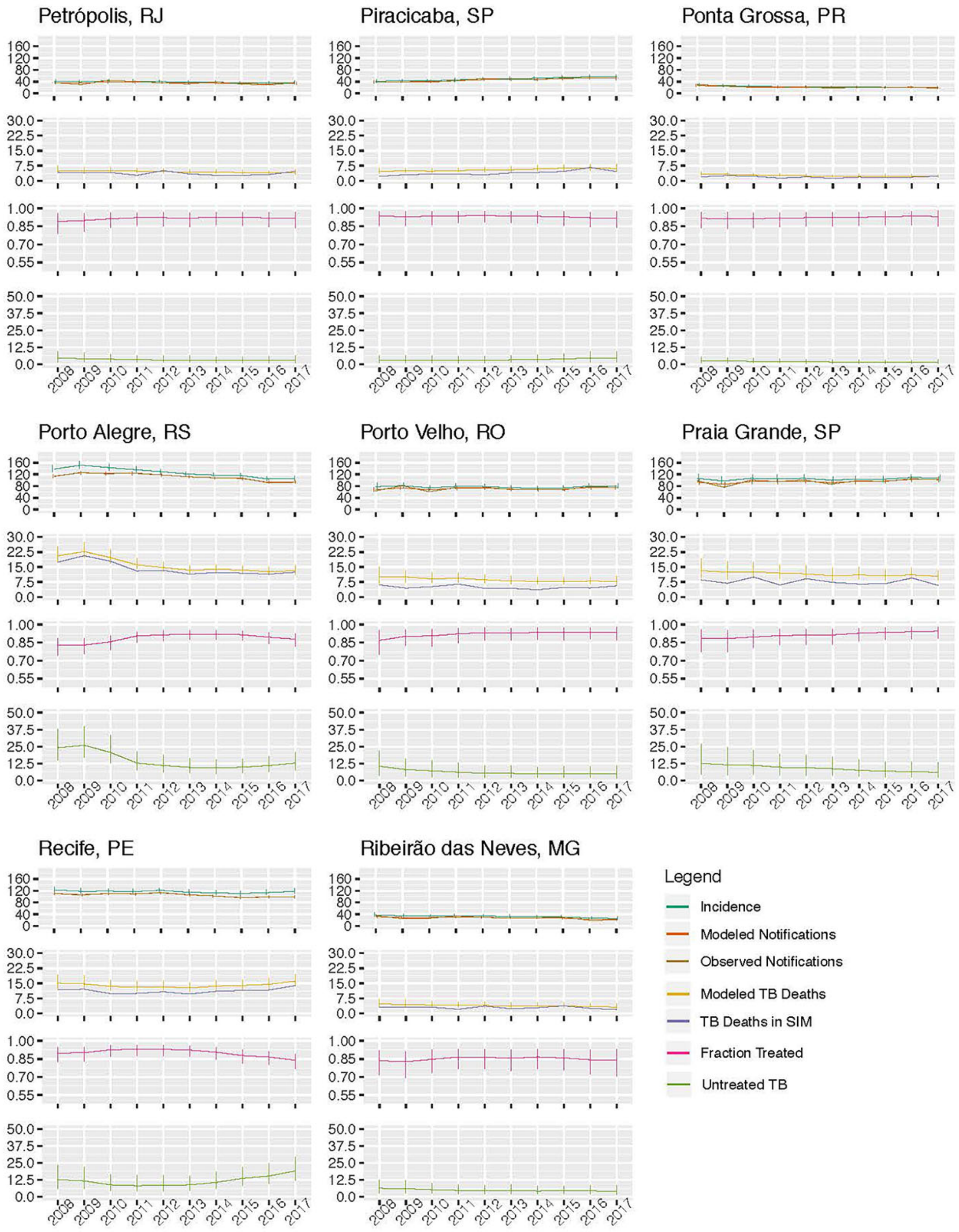

Figure S2: Trends in Observed and Modeled TB Burden by Municipality, 2008 – 2017

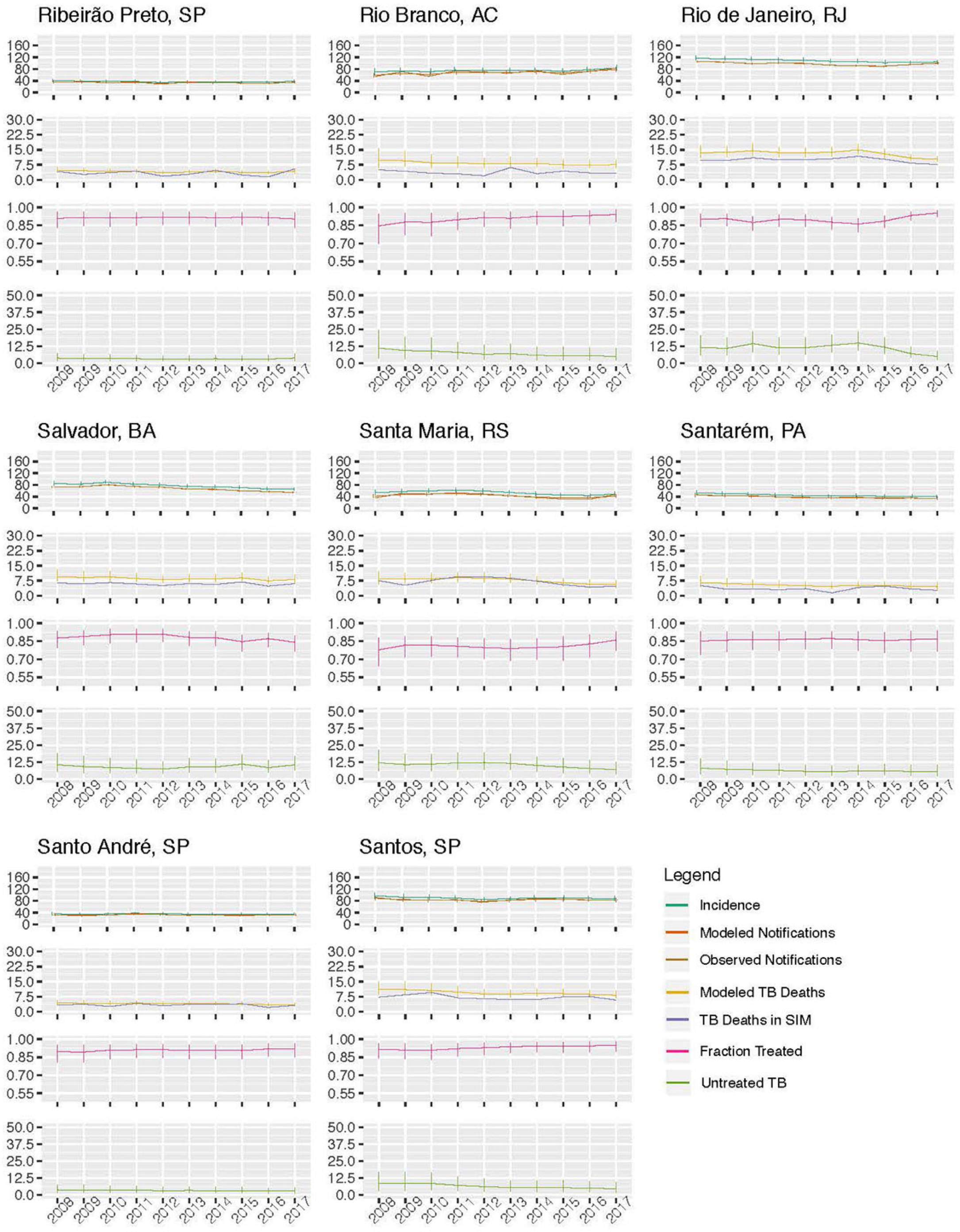

Figure S2: Trends in Observed and Modeled TB Burden by Municipality, 2008 – 2017

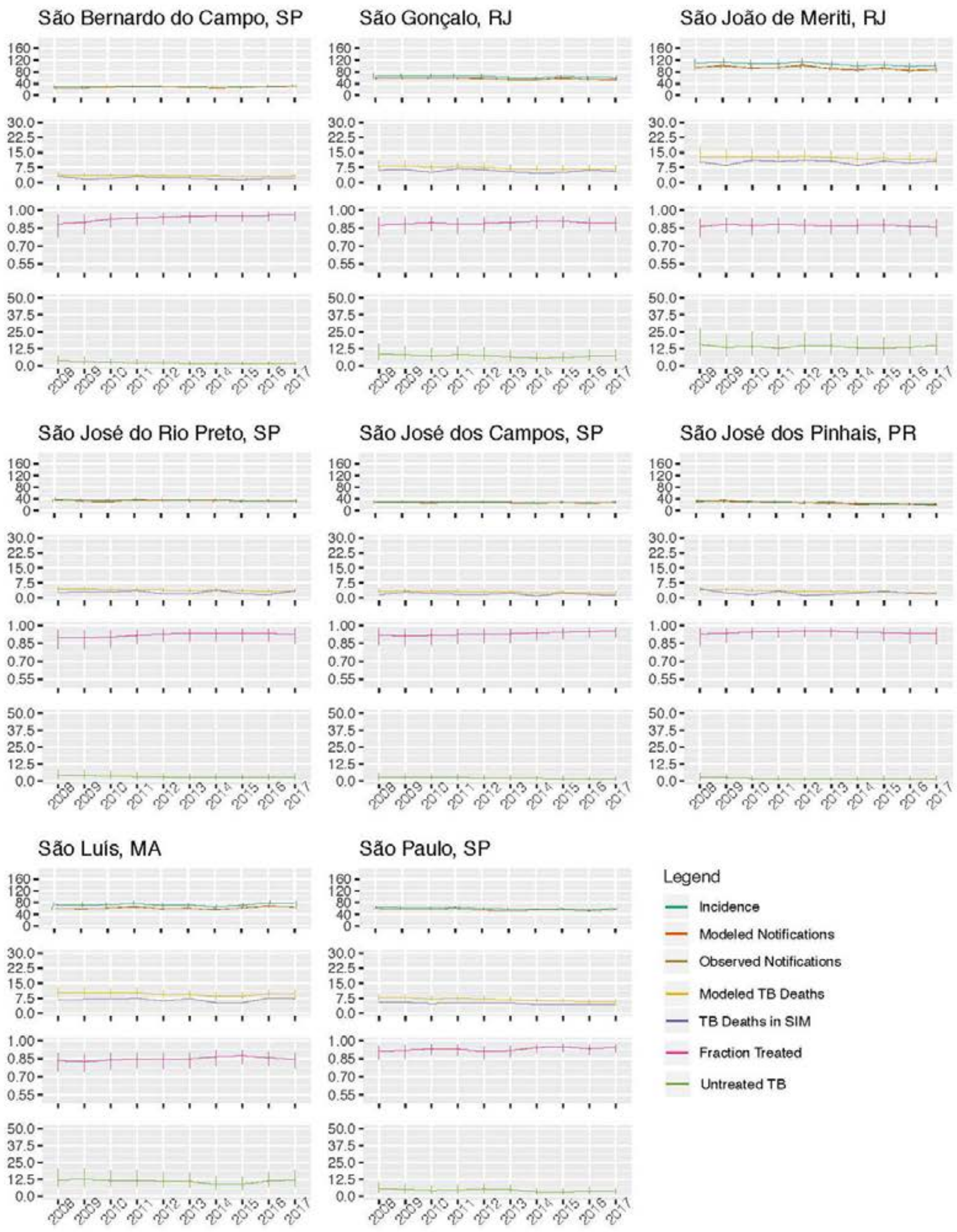

Figure S2: Trends in Observed and Modeled TB Burden by Municipality, 2008 – 2017

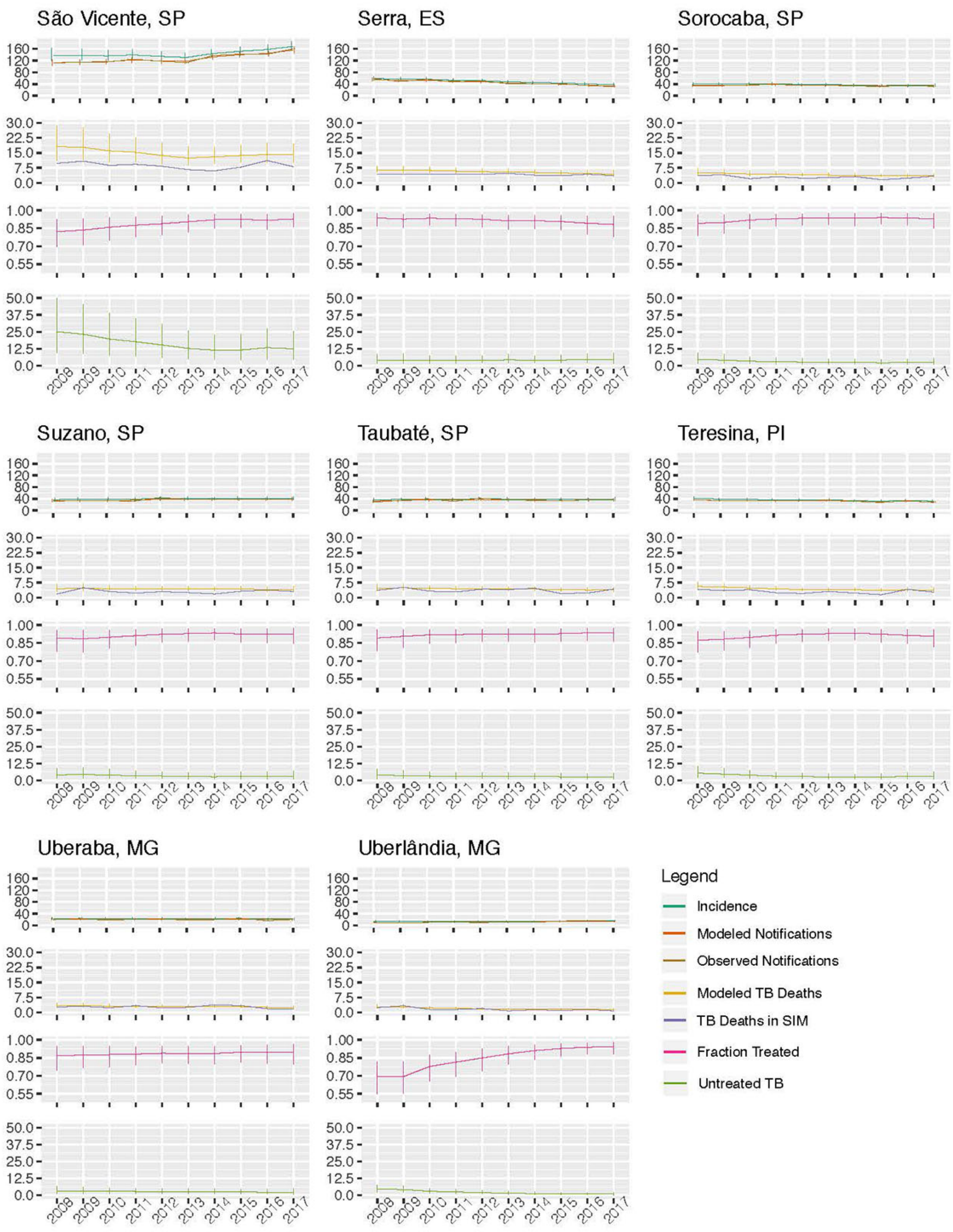

Figure S2: Trends in Observed and Modeled TB Burden by Municipality, 2008 – 2017

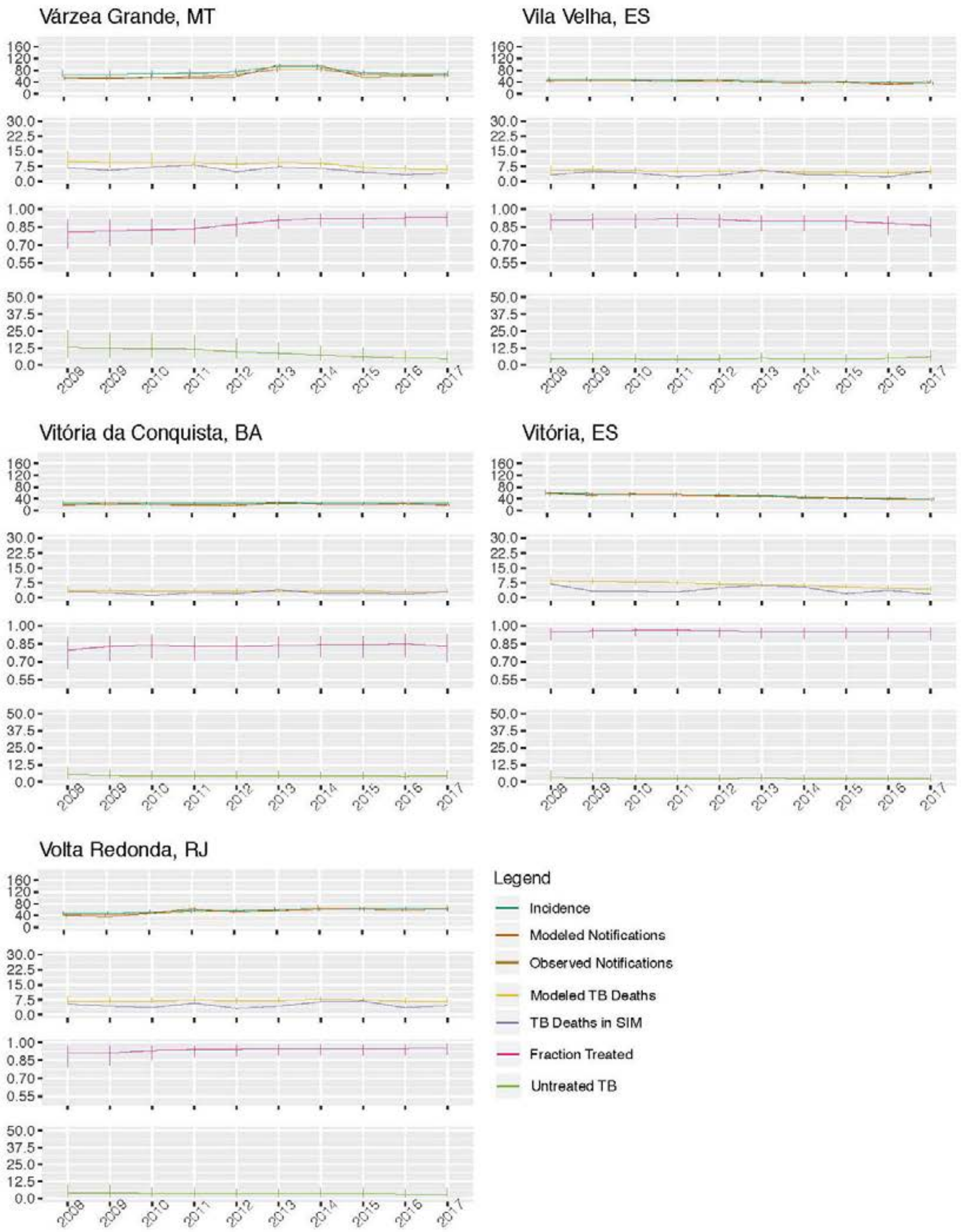

Appendix Figure 2. Trends in observed and modeled TB burden by municipality, Brazil, 2008–2017.

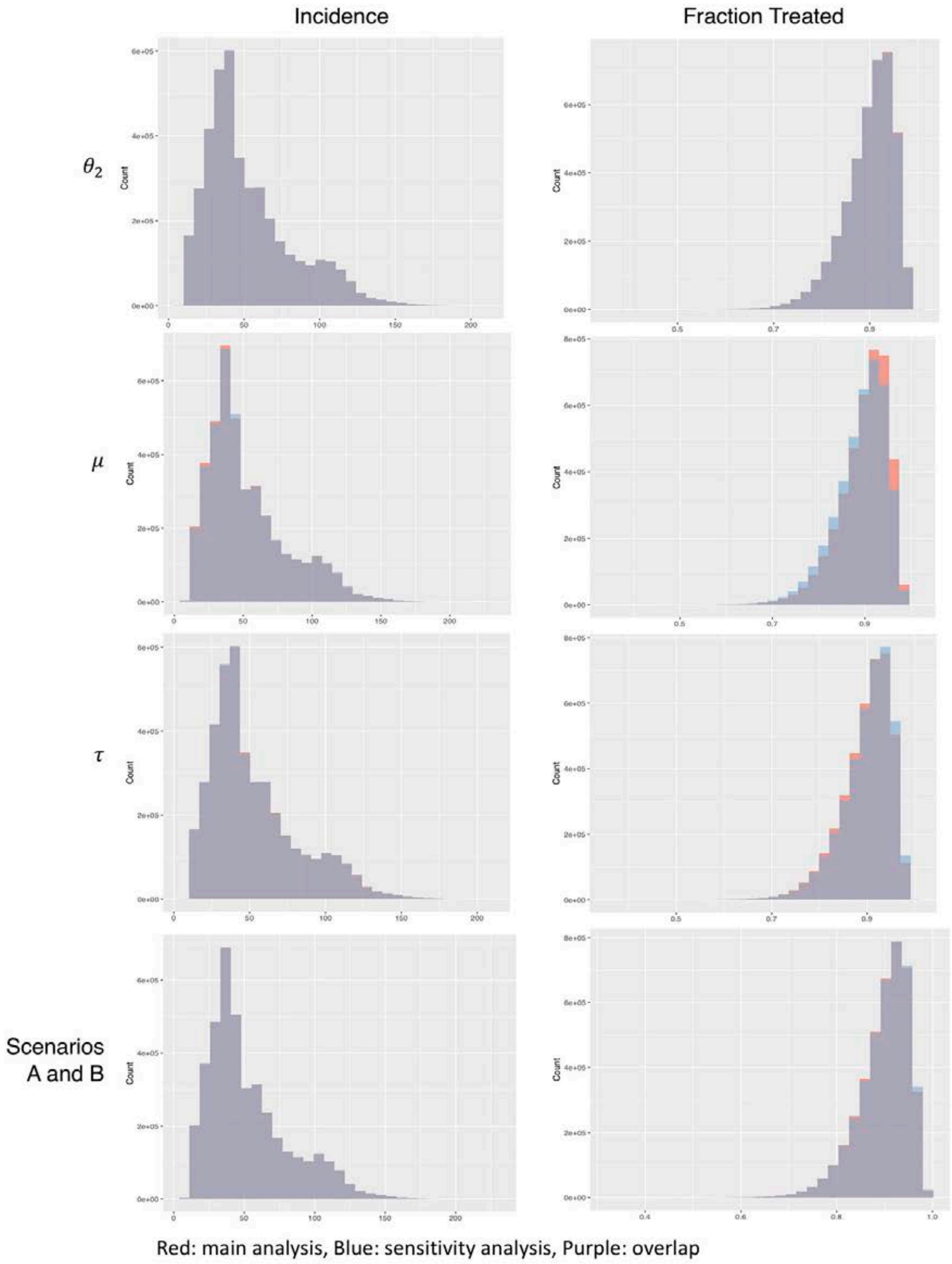

Appendix Figure 3. Sensitivity analysis of TB burden by municipality, Brazil, 2008–2017. Red indicates main analysis; blue, sensitivity analysis. Purple shows overlap of data between the analyses.
